# Using a Prime-Boost Vaccination Strategy That Proved Effective for High Resolution Epitope Mapping to Characterize the Elusive Immunogenicity of Survivin

**DOI:** 10.3390/cancers13246270

**Published:** 2021-12-14

**Authors:** Robert C. Mould, Jacob P. van Vloten, Amanda W. K. AuYeung, Scott R. Walsh, Jondavid de Jong, Leonardo Susta, Anthony J. Mutsaers, James J. Petrik, Geoffrey A. Wood, Sarah K. Wootton, Khalil Karimi, Byram W. Bridle

**Affiliations:** 1 Department of Pathobiology, University of Guelph, Guelph, ON N1G 2W1, Canada; rmould@uoguelph.ca (R.C.M.); jvanvlot@uoguelph.ca (J.P.v.V.); aauyeung@notchtx.com (A.W.K.A.); jddejong@viricabiotech.com (J.d.J.); lsusta@uoguelph.ca (L.S.); gewood@uoguelph.ca (G.A.W.); kwootton@uoguelph.ca (S.K.W.); kkarimi@uoguelph.ca (K.K.); 2 McMaster Immunology Research Centre, McMaster University Hamilton, Hamilton, ON L8S 3L8, Canada; walshs8@mcmaster.ca; 3 Department of Biomedical Sciences, University of Guelph, Guelph, ON N1G 2W1, Canada; mutsaers@uoguelph.ca (A.J.M.); jpetrik@uoguelph.ca (J.J.P.)

**Keywords:** cancers, vaccines, epitope mapping, survivin, green fluorescent protein, prime-boost, recombinant virus, rhabdovirus, adenovirus

## Abstract

**Simple Summary:**

The generation of tumor-specific T cells remains a pillar of modern cancer immunotherapy. Exogenous vaccines often rely on targeting tumor-associated antigens. The anti-apoptotic protein survivin has been deemed a high priority target due to its overexpression in a wide variety of tumor types. To support the analysis of tumor-associated T cell responses, optimization of epitope mapping would be valuable. A heterologous prime-boost vaccination strategy was designed to target survivin to induce anti-tumor immune responses. However, survivin-specific T cell responses could not be detected in mice. Potential mechanisms to explain this failure were explored. To confirm the robustness of the vaccination platform, enhanced green fluorescent protein (eGFP) was targeted since it has been defined as a protein with relatively low immunogenicity. In this context the vaccination strategy uncovered novel T cell epitopes from eGFP in two strains of mice. This research highlighted the utility of the vaccine platform to triage potential target antigens based on their immunogenicity.

**Abstract:**

Survivin is a member of the inhibitor of apoptosis family of proteins and has been reported to be highly expressed in a variety of cancer types, making it a high priority target for cancer vaccination. We previously described a heterologous prime-boost strategy using a replication-deficient adenovirus, followed by an oncolytic rhabdovirus that generates unprecedented antigen-specific T cell responses. We engineered each vector to express a mutated version of full-length murine survivin. We first sought to uncover the complete epitope map for survivin-specific T cell responses in C57BL/6 and BALB/c mice by flow cytometry. However, no T cell responses were detected by intracellular cytokine staining after re-stimulation of T cells. Survivin has been found to be expressed by activated T cells, which could theoretically cause T cell-mediated killing of activated T cells, known as fratricide. We were unable to recapitulate this phenomenon in experiments. Interestingly, the inactivated survivin construct has been previously shown to directly kill tumor cells in vitro. However, there was no evidence in our models of induction of death in antigen-presenting cells due to treatment with a survivin-expressing vector. Using the same recombinant virus-vectored prime-boost strategy targeting the poorly immunogenic enhanced green fluorescent protein proved to be a highly sensitive method for mapping T cell epitopes, particularly in the context of identifying novel epitopes recognized by CD4^+^ T cells. Overall, these results suggested there may be unusually robust tolerance to survivin in commonly used mouse strains that cannot be broken, even when using a particularly potent vaccination platform. However, the vaccination method shows great promise as a strategy for identifying novel and subdominant T cell epitopes.

## 1. Introduction

The induction and/or enhancement of tumor-specific T cell responses remains a pillar of modern cancer immunotherapy but is accompanied by many challenges [1]. When patients lack a pre-existing tumor-specific T cell repertoire, it can be difficult to find a suitable antigen to target due to the plethora of cancer types, as well as the vast heterogeneity of cells within a tumor. As such, there has been a large body of research dedicated to finding ideal target antigens with as broad an indication as possible. The prioritization of candidate antigens has been summarized previously [2]. Survivin (also known as baculoviral inhibitor of apoptosis repeat containing 5 [BIRC5]), is a member of the inhibitor of apoptosis family, and ranks highly among potential targets due to its overexpression in a wide variety of cancers [3]. Moreover, expression of survivin in cancer cells is associated with resistance to radio- and chemo-therapies, implying that the elimination of survivin-expressing cells could induce selection pressure toward cells that are amenable to conventional treatment options [4]. In addition to being highly expressed in an array of cancer types, survivin is also expressed in many tissues during fetal development [3]. However, after birth, its expression is downregulated in normal tissues. Although originally thought to be largely absent in all normal tissues, it can be upregulated on some terminally differentiated cells under conditions of hypoxia and/or inflammation [5,6]. Additionally, neutrophils are capable of upregulating survivin in response to cytokine signaling [7]. However, due to its high priority as a tumor antigen, we sought to extend the recombinant virus vaccine platform we have described previously, which uses a replication deficient adenovirus as a primary vaccine followed by a rhabdovirus-vectored boost, to target murine survivin in pre-clinical mouse models [8]. There have been several clinical cancer immunotherapy studies that targeted survivin in humans with defined human leukocyte antigen (HLA) class I-binding peptides [9,10,11]. To our knowledge, no group has been able to fully characterize a survivin-specific T cell epitope map using overlapping peptides in C57BL/6 or BALB/c mice, which we established as the first priority. Of note, however, two groups found that vaccination against the murine survivin_53–67_ (DLAQCFFCFKELEGW) peptide was able to induce a T cell response in C57BL/6 mice [12,13]. Both groups reported that vaccination against this 15-mer peptide (or a smaller peptide within the 15-mer) induced anti-tumor responses. These immune responses were characterized using cell-mediated cytotoxicity assays; an interferon (IFN)-γ-detecting enzyme-linked immune absorbent spot (ELISpot) or an MHC class I of the murine ‘b’ haplotype (H-2K^b^) peptide-pulsed dimer staining. Therefore, we deemed it reasonable to clone full-length survivin with a single amino acid change (threonine to alanine at position 34 [T34A]) into viral vaccine vectors. The mutated version of survivin was used to inhibit phosphorylation and ensure that any cells infected with the vectors could not be accidentally immortalized [14]. Further, this mutated form of survivin was previously found to have anti-cancer properties when added into several cancer cell lines in in vitro and in vivo models, while notably sparing healthy cells [15,16].

We previously developed a potent vaccination strategy for priming and boosting T cell responses against a transgene-encoded antigen expressed from two viral vectors [8]. Specifically, the priming vector was an E1/E3-deleted replication-deficient human serotype 5 adenovirus administered intramuscularly. The secondary vaccine vector was a replication-competent vesicular stomatitis virus (VSV) delivered intravenously. The replication-deficient adenovirus proved to be ideal for priming a T cell response against self-derived tumor antigens by virtue of focusing the response on the transgene-encoded antigen that was expressed at relatively high concentrations when under the control of a strong promoter. In contrast, T cell responses against the non-replicating viral backbone were relatively weak. Using a heterologous boost with VSV resulted in a secondary T cell response against the tumor antigen. Notably, this secondary T cell response was massive and could be achieved after a very short prime-boost interval by driving antigen presentation within an immunoprivileged site in the spleen [17]. Thus, a recombinant adenovirus expressing the mutated murine survivin (Ad-T34A-mSurvivin), as well as a Maraba virus (MG1-T34A-mSurvivin) were engineered. In the original iteration of our prime-boost strategy, a human serotype 5 adenovirus was used as the primary vaccine vector. However, for future avoidance of the high rate of seroprevalence of adenovirus serotype 5-neutralizng antibodies in humans, we switched to a rare recombinant replication-deficient human serotype 48 adenovirus (Ad48) that had been discovered and characterized by Dr. Dan Barouch‘s group [18]. Compared to our original prime-boost platform that utilized VSV as a booster, Maraba virus proved to be significantly more potent at boosting transgene-specific T cell responses [19]. Therefore, we hypothesized that vaccinating mice with Ad-T34A-mSurvivin followed by MG1-T34A-mSurvivin would induce potent survivin-specific T cell responses that could then be evaluated for efficacy in multiple murine cancer models.

## 2. Materials and Methods

### 2.1. Mice

Female specific pathogen-free C57BL/6 and BALB/c (major histocompatibility haplotypes ‘b’ and ‘d’, respectively) mice (Charles River Laboratories, Wilmington, DE, USA), at 36–50 days of age upon arrival, were housed in a controlled environment in the animal isolation unit at the University of Guelph (Guelph, ON, Canada). Food and water were provided ad libitum. Mice were acclimatized to the facility for one week prior to beginning experiments.

### 2.2. Cell Cultures

Human embryonic kidney-293 (HEK293), A549 lung carcinoma, and Vero cells that were originally derived from kidney epithelial cells extracted from an African green monkey (*Chlorocebus* sp.) (all cell lines were from the American Type Culture Collection (ATCC)) were cultured in Dulbecco’s Modified Eagle’s Medium (DMEM) containing 10% heat-inactivated bovine calf serum (GE Healthcare Life Sciences, Chicago, IL, USA). All cells were cultured at 37 °C in a humidified atmosphere with 5% CO_2_ and were confirmed to be mycoplasma-free prior to use (MycoAlert PLUS detection kit, Lonza, TX, USA).

### 2.3. Plasmids and Vector Construction

The human adenovirus serotype 48 genome and adaptor plasmids (pWe.Ad48.pIX-rITR.dE3.5orf6, and pAdApt, respectively) were a generous gift from Dr. Dan Barouch (Harvard, MA, USA) [18]. The Maraba virus genome plasmid and helper plasmids (pMG1-genome and pCI-Neo-MG1-N, pCI-Neo-MG1-P, pCI-Neo-MG1-G, and pCI-Neo-MG1-L, respectively) were a generous gift from Dr. David Stojdl (Children’s Hospital of Eastern Ontario, Ottawa, ON, Canada) [20]. The pCAGEN plasmid was acquired from Addgene (USA) and was used to clone the cytomegalovirus (CMV) early enhancer/chicken β actin (CAG) promoter [21] in replacement of the CMV promoter in the pAdApt plasmid via the SpeI and EcoRI restriction endonuclease cleavage sites. RNA containing murine survivin mRNA was extracted from TRIzol (Invitrogen, Waltham, MA, USA)-treated murine B16-F10 melanoma cell (ATCC) lysates following the manufacturer’s instructions. cDNA was generated from the extracted RNA using qScript cDNA SuperMix (Quantabio, Beverly, MA, USA) by following the product instructions. A PCR reaction with the cDNA template was performed with the primers listed in Supplementary Table S1 to create a FLAG-tagged mSurvivin insert, which was cloned into the pAdApt vector using KpnI (5′) and BamHI (3′) cleavage sites. Similarly, enhanced green fluorescent protein (eGFP) was cloned into pAdApt using EcoRI (5′) and BamHI (3′) cleavage sites to create a new eGFP vector. The pAdApt-mSurvivin vector was used to create an inactive survivin mutant by switching the threonine at position 34 to alanine via site-directed mutagenesis with the primers found in Supplementary Table S1 (pAdApt-T34A-mSurvivin). The pAdApt-T34A-mSurvivin vector DNA was used as a PCR template to amplify the mutated survivin transgene with a Myc-tag in place of the FLAG-tag, as well as SalI and SpeI cleavage sites using primers listed in Supplementary Table S1. This product was cloned into the MG1-genome plasmid between the G and L protein genes (pMG1-T34A-mSurvivin). All engineered changes were confirmed via Sanger sequencing prior to cloning at the Genomics Facility, University of Guelph.

### 2.4. Viruses

Replication-deficient E1/E3-deleted, recombinant human Ad48, expressing mutated murine survivin (Ad-T34A-mSurvivin) was rescued using the pWe.Ad48.pIX-rITR.dE3.5orf6 and pAdApt-T34A-mSurvivin plasmids, and propagated as described previously [18]. Briefly, propagation was done in HEK-293 cells and purified by ultracentrifugation on a cesium chloride gradient. A second Ad48 vector, expressing eGFP (Ad-eGFP) was rescued and propagated similarly. Both vectors were plaque-purified three times in HEK293 cells prior to scale-up. The Maraba virus vector MG1-T34A-mSurvivin was rescued in A549 cells by plasmid transfection and co-infection with recombinant modified vaccinia virus Ankara expressing a T7 polymerase (MVA-T7), as described previously [20]. The vector was triple plaque-purified, propagated, and titered in Vero cells. VSV-eGFP has been described previously [22]. For the cytotoxicity and co-vaccination experiments, Ad-hDCT and Ad-SIINFEKL were used and have been described previously [23]. Ad-hDCT expresses the melanoma-associated antigen human dopachrome tautomerase (hDCT) [24]. Ad-SIINFEKL expresses luciferase, as well as the CD8^+^ immunodominant epitope to chicken ovalbumin in C57BL/6 mice (OVA_257–264_).

### 2.5. Vaccination Protocol

Mice were vaccinated with a total dose of 1 × 10^8^ infectious units of Ad48 via intramuscular injection in both hind limbs, as described previously [25]. Maraba virus and VSV were administered intravenously in 200 μL of phosphate-buffered saline (PBS) at 1 × 10^9^ plaque-forming units (pfu). Control groups were not vaccinated.

### 2.6. Sample Processing

Blood sampled from the retro-orbital sinus was heparinized to prevent clotting, and the volumes were recorded. Samples were kept on ice during transportation and processing. Single-cell suspensions were made by pressing spleens between the frosted ends of glass microscope slides. Erythrocytes in blood and spleens were lysed. Cells were counted using an improved Neubauer counting chamber using trypan blue dye exclusion to assess viability, which was consistently >90%. All cells were plated in Roswell Park Memorial Institute (RPMI)-1640 medium containing 10% heat-inactivated bovine calf serum, 0.1% 2-mercaptoethanol, and penicillin/streptomycin (cRPMI; GE Healthcare Life Sciences) in 96-well round-bottom plates and were maintained at 4 °C during handling and centrifugation (500× *g*). Up to two million cells were added to each well of a 96-well plate. Samples were processed by the same individual to reduce technical variability.

### 2.7. Quantification of T Cells by Flow Cytometry

Blood- and spleen-derived cells were re-stimulated by adding transgene-derived peptides to a final concentration of one µg/mL. Controls were unstimulated. After one hour, brefeldin A (ThermoFisher Scientific, Waltham, MA, USA) was added to each sample to block golgi apparatus-mediated export of proteins. After another four hours, cells were centrifuged, re-suspended in PBS + 1% bovine serum albumin and stained with antibodies. Fc receptors were blocked with anti-CD16/CD32 (clone 93; BD Biosciences, Mississauga, ON, Canada) for 20 min. Surface staining for 20 min was performed with anti-CD3, anti-CD4, and anti-CD8 (clones 145-2C11, RM4-4, and 53–6.7, respectively; BD Biosciences). A fixable viability dye stain (ThermoFisher Scientific) was used to ensure that only live cells would be included in analyses. Following treatment with Intracellular Fixation Solution (ThermoFisher Scientific) for 20 min, intracellular staining was done for 20 min using anti-TNF-α and anti-IFN-γ in a permeabilization solution (clones MP6-XT22 and XMG1.2, respectively; ThermoFisher Scientific). Stained cells were suspended in 200 μL of PBS + 1% bovine serum albumin, filtered through a mesh with 70 μm pores, and analyzed using a FACS Canto II flow cytometer (Becton Dickinson) using FACS Diva Software Version 8 for data acquisition. Analysis was completed with FlowJo Software version 10 (FlowJo LLC, Ashland, OR, USA).

### 2.8. Peptides

For quantification of survivin-specific and eGFP-specific T cell responses, cells were re-stimulated ex vivo with overlapping peptide libraries, with one 15-mer peptide per sample (PepScan Systems, Lelystad, The Netherlands). A complete list of peptides can be found in Supplementary Tables S2 and S3.

### 2.9. Western Blotting

Infected cell lysates were prepared using Vero cells seeded into T25 cm^2^ flasks, which were infected at a multiplicity of infection (MOI) of 10 and 1 for adenovirus and Maraba virus, respectively. The infection was started in one mL of media, incubated for one hour, and subsequently topped up to five mL. Cells were pelleted 24 h later and lysed by resuspending in radioimmunoprecipitation assay buffer (RIPA) containing a protease inhibitor cocktail (Sigma-Aldrich, St. Louis, MO, USA) for 30 min on ice. Clarified lysates were obtained by centrifugation at ~18,000× *g* for 30 min. Approximate protein concentrations of the lysates were determined using a Bradford assay. The proteins within the lysates were separated by sodium dodecyl sulfate polyacrylamide gel electrophoresis (SDS-PAGE) and transferred onto a 0.2 µm polyvinylidene membrane (PVDF). The membranes were blocked at 4 °C overnight using a blocking solution as specified by the manufacturer for each of the primary antibodies, which were in either 5% *w*/*v* skim milk in PBS + 1% Tween 20 (PBST) or 5% *w*/*v* bovine serum albumin (BSA) in PBST. Primary antibody staining for Myc (9B11, Cell Signaling, Danvers, MA, USA), FLAG (D6W5B, Cell Signaling), and survivin (J.33.5, ThermoFisher Scientific, USA) was performed at a 1:1000 dilution in their corresponding blocking solutions at 4 °C overnight. The membranes were washed three times for 15 min on a shaker with PBST, then subsequently incubated with horseradish peroxidase (HRP)-conjugated secondary antibodies in blocking solution for one hour at room temperature at a 1:5000 dilution. Proteins were detected after membranes were treated with Western lightning chemiluminescence substrate (PerkinElmer Health Sciences Canada, Woodbridge, ON, Canada). Images were obtained using a ChemiDoc MP Imaging System (Bio-Rad Laboratories, Mississauga, ON, Canada) and Image Lab software (Bio-Rad, Canada).

### 2.10. In Vivo Cytotoxicity Assay

Syngeneic splenoctyes from female C57BL/6 mice were harvested, processed into single-cell suspensions, and erythrocytes were lysed. Cells were passed through 100 µm filters, resuspended in cRPMI, and live cells were counted using a Neubauer counting chamber and trypan blue dye exclusion. The splenocytes were divided into two groups and labelled with 0.2 µM or 1 µM of Violet Proliferation Dye (VPD) (BD Horizon; Cambridge, UK). One group of splenocytes was pulsed with 10 µg/mL of OVA_257–264_, and the other was pulsed with 10 µg/mL of murine Survivin_53–67_, which are known to bind to C57BL/6 MHC class I [26]. Pulsing was done for one hour at 37 °C. Equal numbers of cells from each group were combined and were washed twice with PBS. Ten million of the labelled splenocytes were injected intravenously into vaccinated mice. Blood and spleen samples were collected six hours post-injection. All samples had their red blood cells lysed, were suspended in 200 μL of PBS + 1% bovine serum albumin, filtered through a mesh with 70 μm pores, and analyzed using a FACS Canto II flow cytometer using FACS Diva Software Version 8 for data acquisition. Analysis was completed with FlowJo Software version 10.

### 2.11. In Vitro Infection of Antigen-Presenting Cells (APCs) with Adenovirus

Syngeneic bone marrow cells from female C57BL/6 mice were harvested and processed into single-cell suspensions. Cells were passed through 100 μm filters, resuspended in cRPMI, and live cells were counted using a Neubauer counting chamber and trypan blue dye exclusion. A heterogeneous population of APCs were generated as described previously [27]. Briefly, three million cells were seeded in a T75 cm^2^ flask in cRPMI containing 20 ng/mL of granulocyte-macrophage colony-stimulating factor (GM-CSF) at time zero. Interleukin (IL)-4 was introduced to the cultures on day two at a concentration of 10 ng/mL. Media and cytokines were refreshed on days two and five. On day seven cells were counted and seeded at 250,000 cells per well in a 96-well plate for each timepoint. Ad-eGFP and Ad-T34A-mSurvivin were serially diluted and added to the cells at MOIs of 0 (media only), 0.01, 0.1, 1, 10, and 100. At the indicated timepoint, the cells were washed and stained with 7-aminoactinomycin D (7AAD) viability dye and analyzed for viability, as well as expression of eGFP by flow cytometry.

### 2.12. In Vivo Immunological Checkpoint Inhibition with Anti-Cytotoxic T Lymphocyte-Associated Protein 4 (CTLA4)

CTLA4-blocking antibodies were obtained from the supernatant of UC10-4F10-11 hybridoma cells (ATCC, Rockville, MD, USA) and purified using a HiTrap protein G column (GE Healthcare Life Sciences) following the manufacturer’s instructions. The purified antibodies were dialyzed against PBS (ThermoFisher Scientific). Antibody concentration and purity was determined with a Bradford assay and SDS-Page Coomassie Blue staining, respectively (Supplementary Figure S4). Mice were given 400 µg of anti-CTLA4 intraperitoneally 24 h prior to vaccination with Ad48-mSurvivin-T34A, and 200 µg was also given 10 min prior to vaccination.

### 2.13. Statistics

Graphing and statistical analyses were done with GraphPad Prism version 10. Graphs show means and standard errors. Frequencies and numbers of T cells in blood and spleens were assessed by one-way analysis of variance with Tukey’s multiple comparisons test. Numbers of blood-derived cells were normalized based on sample volumes. All numbers were expressed as relative values to account for the inevitable loss of cells during processing and to prevent direct inference of actual physiological numbers. One person processed each set of samples and was blinded to treatment groups. Differences between treatments were considered significant at *p* ≤ 0.05.

## 3. Results

### 3.1. Murine Survivin Was Successfully Tagged, Mutated, Cloned into, and Then Expressed by Adenovirus and Maraba Virus Vectors

**Figure 1:**
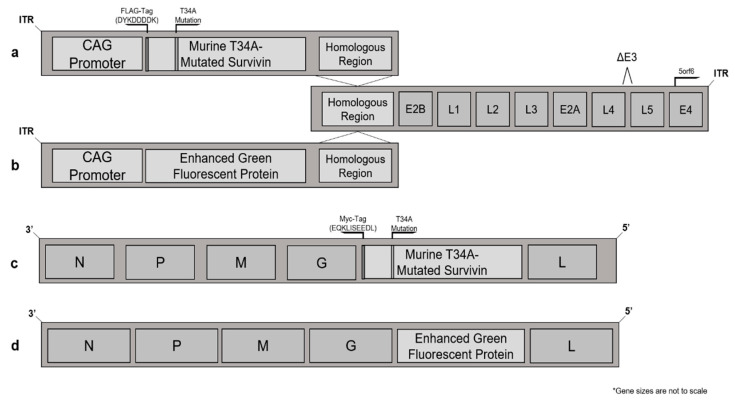

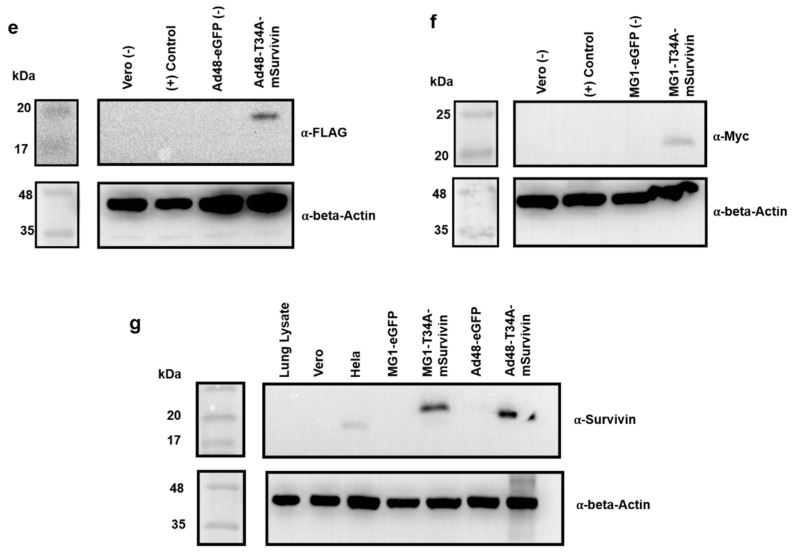
Schematic representation of murine survivin- and enhanced green fluorescent protein (eGFP)-expressing virus vectors, with Western blots showing expression of survivin after infecting cells. (**a**) The adenoviral survivin adaptor region for Ad48-T34A-mSurvivin expressed the mutated survivin transgene under a CAG promoter, which was FLAG-tagged for ease of determining expression levels and distinguishing the transgene from endogenous survivin. This was recombined with the adenoviral genome at the homologous region noted in place of the E1 protein. (**b**) Similarly, an eGFP-expressing adaptor region of the Ad48-eGFP virus recombined with the homologous region of the adenoviral genome. (**c**) The Maraba virus mutated-survivin vector MG1-T34A-mSurvivin had a Myc tag and was expressed between the G and L proteins of the Maraba virus genome. (**d**) The VSV-eGFP vector was generated by cloning the eGFP between the G and L proteins of the Indiana strain of wild-type VSV. SDS-PAGE Western blots of survivin-expressing vectors were generated using infected cell lysates. (**e**) Vero cells were infected with recombinant Ad48 vectors for 24 h. Uninfected Vero cell-derived lysate was used as a control. All lanes were loaded with 15 µg of lysate and probed with a FLAG tag-specific antibody. (**f**) Lysate from Vero cells 24 h post-infection with recombinant Maraba virus. Lysate from uninfected Vero cells was used as a control. All lanes were loaded with 15 µg of lysate and probed with an anti-Myc tag. (**g**) Vero cells infected with either Ad48 vectors, Maraba virus vectors or uninfected negative controls. All lanes were loaded with 15 µg of lysate and probed with a survivin-specific antibody. Normal lung lysate was used as a negative control that did not express substantial amounts of endogenous survivin. The tagged murine survivin was expected to be ~5.6% larger than the endogenous survivin protein. ITR = inverted terminal repeats.

We utilized two viral vectors to formulate a potent heterologous prime-boost vaccine strategy targeting survivin. Murine survivin was engineered to be expressed in both adenovirus and Maraba virus vectors using primer sets outlined in Supplementary Table S1 and represented schematically in Figure 1. The adenovirus adaptor plasmid, which contained the expression sequences of the survivin transgene, had its CMV promoter switched to a CAG promoter to enhance gene expression [28]. The survivin transgene was also tagged using a FLAG-tag sequence in the adenoviral vector (Figure 1a). Similarly, the survivin transgene was Myc-tagged in place of the FLAG-tag in the Maraba virus vector to avoid any risk of boosting any potential immune responses against the FLAG-tagged portion of the survivin protein derived from the Ad48. Myc-tagged survivin was cloned into the Maraba virus genome between the G and L proteins (Figure 1b). The survivin transgenes were mutated at position 34, where a threonine was changed to an alanine via site-directed-mutagenesis using primers outlined in Supplementary Table S1. This change has previously been described to inactivate the anti-apoptotic properties of the survivin protein [29]. eGFP was cloned into the same positions and in place of survivin in each vector, without mutations or tags, to generate Ad-eGFP (Figure 1c).

Viral gene expression of survivin was determined by Western blotting using lysates from infected cells (Figure 1e,f). Wild-type murine survivin bands were expected to be ~16.5 kDa in size [30]. However, the virally expressed survivin transgenes were slightly larger due to the addition of tags at the N-terminus of the proteins. Indeed, this size difference was confirmed by the endogenous expression of survivin within HeLa cells (positive control), which was smaller than the recombinant vector bands (Figure 1g). Virus-produced survivin could be differentiated from endogenous survivin in cancer cell lines using the FLAG and Myc tags. The expression of survivin from adenovirus and Maraba virus vectors was also confirmed using survivin-specific antibodies. Additional confirmatory blots, as well as the uncropped blots, can be found in Supplementary Figure S2.

### 3.2. Vaccination against Murine Survivin Was Unable to Induce a Survivin-Specific Cytotoxic T Cell Response

**Figure 2:**
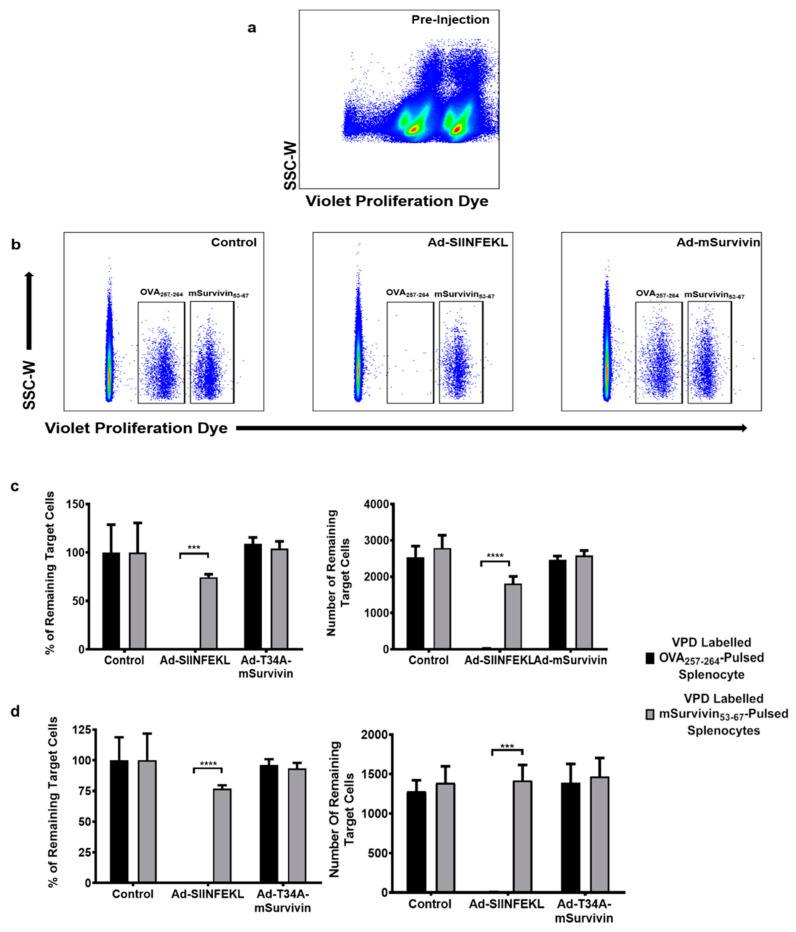
Vaccination against murine survivin was unable to induce a survivin-specific cytotoxic response. (**a**) Splenocytes from female C57BL/6 mice were split in two groups and stained with violet proliferation dye (VPD) at a concentration of 0.2 µM or 1 µM and pulsed with either OVA_257–264_ (SIINFEKL, VPD^lo^) or mSurvivin_53–67_ (DLAQCFFCFKELEGW, VPD^hi^) peptides. (**b**) Both sets of VPD-labelled target cells were injected into Ad-SIINFEKL-vaccinated, Ad48-T34A-mSurvivin-vaccinated, and control mice 10 days post-immunization. The adenovirus-treated mice were given 1 × 10^8^ infectious units intramuscularly. The SIINFEKL-pulsed target cells were eliminated in the Ad-SIINFEKL-vaccinated mice, and were unaffected in the control, and survivin-vaccinated groups. mSurvivin_53–67_ peptide-pulsed targets cells were not killed in the survivin-vaccinated group, nor the Ad-SIINFEKL-vaccinated or control groups. Percent of remaining (left) and total recoverable number (right) target cells graphically illustrate a cytotoxic immune response to SIINFEKL in the Ad-SIINFEKL-vaccinated group in the blood (**c**) and spleens (**d**). Survivin-specific target cells were not eliminated in any group. Two-way analysis of variance with Tukey’s multiple comparison test determined significance for the graphs showing the frequency (left) and number (right) of remaining VPD-labelled target cells. The frequencies were normalized to the recovery frequency values of each target subset in unvaccinated mice. *** = *p* ≤ 0.001; **** = *p* ≤ 0.0001; SSC-W = side scatter-width.

Following the successful rescue and propagation of the murine survivin-expressing viral vectors, the first objective was to generate a complete epitope map of full-length murine survivin. Surprisingly, however, intracellular cytokine staining after re-stimulation with the peptide library failed to reveal any T cell responses above background after the prime-boost vaccination protocol in both BALB/c and C57BL/6 mice (Supplementary Figure S3). To simplify troubleshooting, we opted to use a known epitope in C57BL/6 mice, mSurvivin_53–67_ (DLAQCFFCFKELEGW) [12,13], to evaluate CD8^+^ T cell responses. It has previously been reported that activated T cells upregulate survivin, and this could hypothetically induce fratricide [31,32]. If true, this would generate a self-defeating T cell response, where newly activated survivin-specific T cells upregulate survivin and eliminate each other. We explored this in the context of an in vivo cytotoxicity assay, where C57BL/6 mice were injected with Ad-T34A-mSurvivin (at 1 × 10^8^ infectious units (ifu) intramuscularly), Ad-SIINFEKL, or PBS (control). Ten days later, syngeneic donor splenocytes were labelled with high or low concentrations of VPD. The VPD^hi^ splenocytes were pulsed with the survivin-specific peptide (mSurvivin_53–67_), and the VPD^lo^ splenocytes were pulsed with the OVA_257–264_ peptide. These cells were combined in a 1:1 ratio and injected intravenously into the vaccinated and control groups. A pre-injection sample was taken and assessed to ensure proper VPD-labelling (Figure 2a). Six hours post-injection, splenocytes were recovered from the Ad-T34A-mSurvivin-immunized and PBS-treated control groups, where no statistically significant change was detected in the ratio of VPD^hi^:VPD^lo^ cells (Figure 2b). This suggested there had been no killing of survivin-specific cells in mice that received the survivin-targeting vaccine. Conversely, the SIINFEKL-pulsed VPD^lo^ splenocytes were eliminated in the blood (Figure 2c) and spleens (Figure 2d) of mice vaccinated with Ad-SIINFEKL, confirming that the vaccine platform was functional. These outcomes provide evidence that vaccination against survivin was unable to generate a cytotoxic T cell response targeting survivin-expressing cells in C57BL/6 mice. However, it remained unclear whether this was due to poor immunogenicity of the survivin transgene or a self-defeating fratricidal effect of newly activated, survivin-expressing, survivin-specific T cells. To test the latter hypothesis, a co-vaccination strategy was used.

### 3.3. Co-Vaccination with Ad-T34A-mSurvivin and Ad-DCT Did Not Dampen Nor Eliminate DCT-Specific T Cell Responses

**Figure 3:**
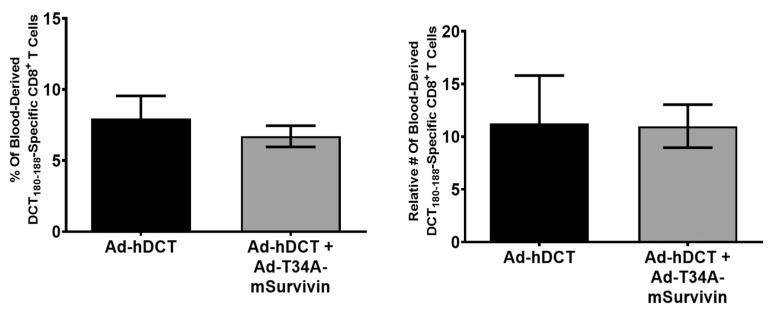
Co-vaccination against survivin and dopachrome tautomerase did not reduce the magnitude of a DCT-specific CD8^+^ T cell response when compared to DCT vaccination alone. Female C57BL/6 mice were vaccinated with Ad-hDCT, Ad-T34A-mSurvivin, or Ad-hDCT + Ad-T34A-mSurvivin at a total dose of 1 × 10^8^ ifu intramuscularly. T cell responses were quantified ten days post-vaccination by flow cytometric assessment of intracellular cytokine staining after ex vivo re-stimulation with peptides. Blood-derived DCT_180–188_-specific CD8^+^ T cell response frequency (**left**) and total number (**right**) were not significantly different in mice vaccinated against DCT alone versus co-vaccination against DCT and murine survivin. Blood-derived mSurvivin_53–67_ specific CD8^+^ T cell responses were not detectable (data not shown). Significance was determined by one-way analysis of variance with Tukey’s multiple comparison test (*n* = 4/treatment).

To evaluate if newly activated T cells were being killed by a survivin-specific response, C57BL/6 mice were vaccinated with Ad-T34A-mSurvivin, Ad-DCT, or both viruses simultaneously at a dose of 1 × 10^8^ ifu/vector (meaning that mice receiving both vaccines received a total dose of Ad of 2 × 10^8^ ifu). The rationale for this design was that T cell fratricide directed at newly activated T cells because of them upregulating survivin should be indiscriminate, meaning that recently activated DCT-specific and survivin-specific T cells should be equally susceptible to killing, mediated by the survivin-specific cells. If this were the case, it would be expected that the DCT-specific response in mice co-vaccinated with Ad-DCT and Ad-T34A-mSurvivin should be lower when compared to mice vaccinated with Ad-DCT only. At the peak of the primary response, blood samples from C57BL/6 mice were re-stimulated with either the immunodominant CD8^+^ T cell epitope from DCT (DCT_180–188_) or the mSurvivin_53–67_ peptide and assessed by flow cytometry after intracellular cytokine staining. mSurvivin_53–67_ peptide-specific responses were not detectable in any group (data not shown). Blood-derived DCT_180-188_-specific responses were quantified in each group (Figure 3). The mean frequencies and relative numbers of DCT_180–188_-specific T cells were not significantly different between the Ad-DCT- and the Ad-DCT + Ad-T34A-mSurvivin-vaccinated groups. Therefore, there was no evidence that T cell fratricide had occurred. Instead, the results suggested that the survivin protein was simply poorly immunogenic in nature. As such, the next goal was to use immunological checkpoint inhibition to try to increase survivin-specific T cell responses to detectable levels.

### 3.4. Ad-T34A-mSurvivin Did Not Significantly Impact the Viability of Antigen-Presenting Cells

**Figure 4:**
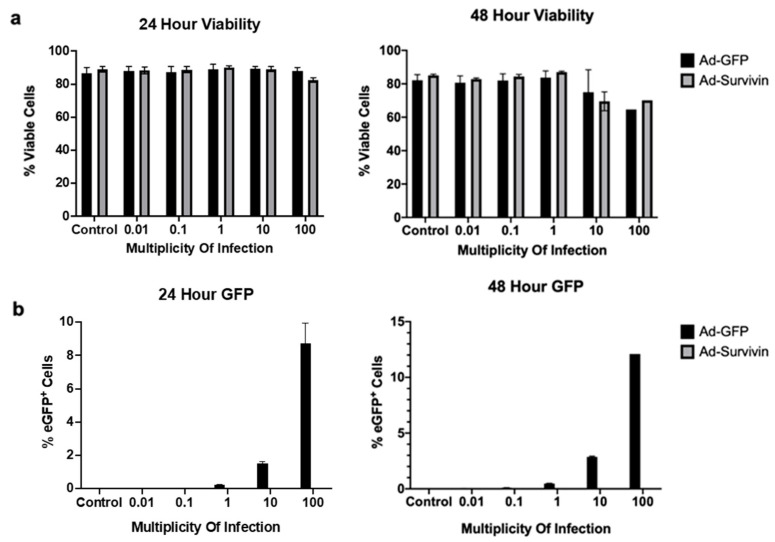
Ad-T34A-mSurvivn was not cytotoxic to antigen-presenting cells. Female C57BL/6 bone-marrow-derived APCs were cultured for seven days with GM-CSF and IL-4. After seven days, the cells were seeded into a 96-well dish at a concentration of 2.5 × 10^5^ cells per well. Each well was treated with either media only (control) or a range of multiplicities of infection with the Ad-eGFP vector or the Ad-T34A-mSurvivin vector. Two timepoints were independently evaluated for (**a**) viability by staining with 7AAD and (**b**) eGFP expression via flow cytometry. Experiments were repeated five times. Means and standard errors are shown.

It has been reported previously that a similar adenoviral construct expressing survivin with a T34A mutation was able to induce apoptosis in a variety of cancer cell lines [16]. Notably, this other research group did not find any cytotoxic effects in normal cells, including smooth muscle cells, which is the targeted site for our vaccine. However, theoretically the specific ablation of APCs by such a vector could constrain any productive vector-specific T cell priming. To address this matter, we cultured a heterogenous pool of APCs from female C57BL/6 murine bone marrow [27] and infected them with a range of doses of adenoviruses expressing T34A-mSurvivin or eGFP as a control (Figure 4). Cells were cultured for either 24 (left panels) or 48 h (right panels) with a multiplicity of infection range of 0 (control), 0.01, 0.1, 1, 10, and 100 for both vectors. (a) At the end of each timepoint, neither the Ad-eGFP or Ad-T34A-mSurvivin vector induced notable toxicities in the bone marrow-derived APCs. (b) Adenoviruses were able to infect APCs at high MOIs when evaluated by eGFP expression mediated by the control vector. The lack of toxicity in vitro matches the safety profile described by other groups and rules out APC-related toxicity as a possible explanation of poor T cell priming from the Ad-T34A-mSurvivin vector.

### 3.5. Antibody-Mediated Blockade of CTLA4 Prior to Priming with Ad-T34A-mSurvivin and Boosting with MG1-T34A-mSurvivin Did Not Unveil Survivin-Specific T Cell Responses

**Figure 5:**
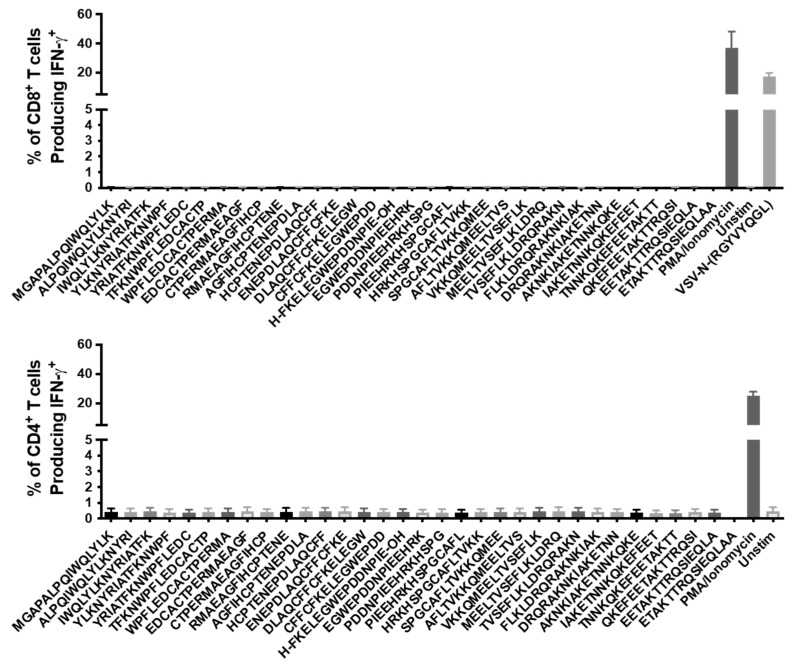
Blocking CTLA4 was unable to rescue the lack of immunogenicity of murine survivin. C57BL/6 mice were given 200 μg of a CTLA4-blocking antibody intraperitoneally 24 h prior to vaccination, as well as 100 μg one hour before vaccination. The mice were then vaccinated with Ad48-T34A-mSurvivin at 1 × 10^8^ ifu and boosted 14 days later with MG1-T34A-mSurvivin at 1 × 10^9^ pfu. Intracellular cytokine staining following peptide re-stimulation at the expected peak of the secondary T cell response failed to reveal detectable survivin-specific CD8^+^ (**top**) or CD4^+^ (**bottom**) T cell responses.

CTLA4 is a receptor found on T cells that provides inhibitory signaling upon binding with costimulatory molecules, such as CD80 and CD86, which are expressed on APCs [33]. CTLA4 blockade is associated with enhanced T cell activation and has emerged as a valuable therapeutic tool for cancers. We sought to use a CTLA4-blocking antibody to enhance the immunogenicity of the survivin vaccines, as this has been reported to help break peripheral tolerance to aberrantly expressed tumor antigens [34]. C57BL/6 mice had their CTLA4 receptors blocked with 400 µg of anti-CLTA4 given intraperitoneally 24 h prior to vaccination with Ad-T34A-mSurvivin, as well as 200 µg just prior to the vaccine being administered. Two weeks later, the mice were boosted with MG1-T34A-mSurvivin, a strategy that has proven to elicit T cell responses of unprecedented magnitudes [8,19], and attempts were made to quantify splenic survivin-specific T cells at the expected peak of the secondary response. An overlapping survivin peptide library was used to re-stimulate splenocytes and intracellular levels of IFN-γ were assessed in CD8^+^ and CD4^+^ T cells via flow cytometry (Figure 5). The results indicated that blocking CTLA4 prior to vaccination against survivin was unable to unveil any obvious survivin-specific T cell responses. The internal positive controls included non-specific stimulation of cells with phorbol 12-myristate 13-acetate (PMA)/ionomycin, as well as a peptide containing a previously described CD8^+^ epitope in C57BL/6 mice from a segment of the VSV N protein (RGYVYQGL) [35], which is also found in the Maraba virus N protein. These control data confirmed the assay was working as intended. This represented the first time that the adenovirus prime and rhabdovirus boost strategy failed to induce robust T cell responses. It had previously proven successful in the context of a variety of antigens.

### 3.6. An Ad Prime Followed by Boosting with VSV Facilitated the Discovery of Novel eGFP Epitopes in C57BL/6 and BALB/c Mice

**Figure 6:**
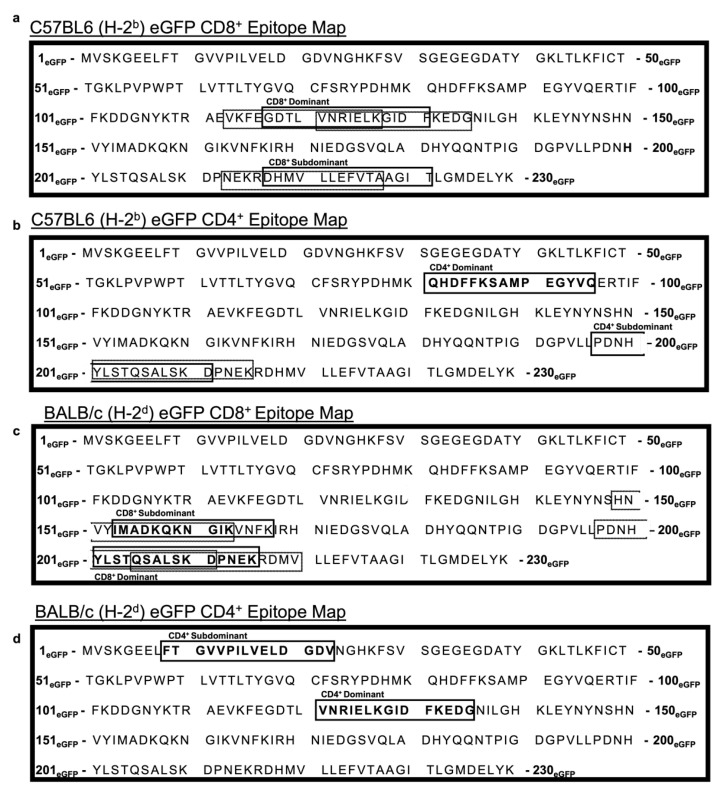
Enhanced green fluorescent protein (eGFP) epitope maps showing dominant and subdominant peptides for CD8^+^ and CD4^+^ T cell responses in C57BL/6 and BALB/c mice. C57BL/6 and BALB/c mice were vaccinated with Ad-eGFP, followed by a VSV-eGFP boost, and eGFP-specific responses were analyzed at the peak of the response (five days post-boost) using peptide re-stimulation and intracellular flow cytometry staining. Shown is the complete eGFP amino acid sequence with boxes that show the peptides that contained the immunodominant and subdominant epitopes for (**a**) CD8^+^ and (**b**) CD4^+^ T cells in C57BL/6 mice. Similarly, peptides containing eGFP-derived (**c**) CD8^+^ and (**d**) CD4^+^ T cell epitopes in BALB/c mice are shown. Sequences containing the complete epitope are boxed with a bolded line. Conversely, sequences containing partial epitopes are boxed in a thin dotted line. Data were derived from a minimum of three mice per group.

To our surprise, the Ad-T34A-mSurvivin prime followed by MG1-T34A-mSurvivin boost was unable to induce detectable T cell responses via intracellular cytokine staining for IFN-γ after a T cell-peptide re-stimulation assay. One possible explanation observed previously might have been T cell-mediated fratricide, where survivin-specific T cells target and eliminate any survivin-expressing cell [31]. Indeed, survivin is expressed in some healthy tissues [36] and has been found to be upregulated on activated T cells [32]. This phenomenon is disputed, however, with a group unable to reproduce this with autologous survivin-specific T cells in which they suspect survivin-related fratricide might be restricted to artificially high avidity TCRs [37]. Moreover, several groups have switched to the use of survivin peptide mimics to improve MHC binding, suggesting that perhaps wild-type survivin is simply poorly immunogenic [38,39]. We therefore deemed it necessary to provide commentary on these matters, as well as determine if our epitope mapping platform had the potential to uncover relatively weak immune responses. To determine the latter, we attempted to determine a full epitope map of eGFP using the same methods used for the survivin experiments. eGFP has been reported previously to be poorly immunogenic in C57BL/6 mice and only slightly more immunogenic in BALB/c mice [40,41]. Using the same adenoviral vector backbone, we generated an Ad-eGFP primary vaccine vector and used vesicular stomatitis virus expressing eGFP (VSV-eGFP) to conduct epitope mapping of eGFP in both mouse strains. To evaluate the potency and utility of our prime-boost vaccine platform considering the lackluster responses targeting survivin, we engineered Ad48 to express eGFP using the same methods used to generate the Ad48-T34A-mSurvivin vector. The immunogenicity of eGFP has generally been poor, particularly in C57BL/6 mice [40,41]. We reasoned that evaluating eGFP-specific T cell responses after vaccination with our routinely used vectors should give us an indication as to how effective they perform when targeting antigens with relatively low immunogenicity. C57BL/6 and BALB/c mice were primed with Ad-eGFP and then boosted 14 days later with wild-type VSV-eGFP (Indiana strain), which is a less potent boosting rhabdoviral vector than the Maraba virus that we had used to target survivin [42]. At the expected peak of the secondary response, spleens were taken and re-stimulated with an overlapping peptide library for eGFP (Supplementary Table S2). The complete epitope map for eGFP for CD8^+^ and CD4^+^ T cell responses in C57BL/6 mice can be found in Figure 6a,b, respectively. Likewise, for BALB/c mice, the CD8^+^ and CD4^+^ T cell epitope maps can be found in Figure 6c,d, respectively. Notably a CD8^+^ T cell epitope in C57BL/6 mice (H-2^b^) was previously defined (DTLVNRIEL) [43]. Our results confirmed this to be an immunodominant epitope based on the response to eGFP_117–131_ (GDTLVNRIELKGIDF). Notably, a weaker T CD8^+^ T cell response was detected against eGFP_217–231_ (DHMVLLEFVTAAGIT). To the best of our knowledge, this subdominant epitope has not been reported previously. With respect to CD4^+^ T cells, we noted a dominant response to eGFP_81–95_ (QHDFFKSAMPEGYVQ). This epitope has been described previously (FKSAMPEGY) [44]. However, a subdominant CD4^+^ epitope was detected within eGFP_197–211_ (PDNHYLSTQSALSKD). Again, to the best of our knowledge, this represents a novel epitope. For BALB/c mice, a CD8^+^ T cell epitope has been elucidated by others (HYLSTQSAL) [45]. Our results demonstrated that a dominant minimal epitope likely existed within eGFP_207–221_(YLSTQSALSKDPNEK). The combination of the previous finding and ours suggests that the peptide “YLSTQSAL” might represent the true minimal epitope, but this would need to be confirmed. As for the C57BL/6 mice, we found a subdominant CD8^+^ T cell epitope within eGFP_153–167_ (IMADKQKNGIKVNFK). We also found dominant and subdominant CD4^+^ T cell epitopes in eGFP_121–135_ (VNRIELKGIDFKEDG) and eGFP_9–23_ (FTGVVPILVELDGDV), respectively. As far as we are aware, these latter three epitopes are novel. Responses to the subdominant CD4^+^ epitope was particularly weak in BALB/c mice. However, the position of IFN-γ^+^TNF-α^+^ cells on dot plots suggested this was an accurate finding (Supplementary Figure S3). These expanded epitope maps could be a useful tool for researchers who want to test the vaccination potential of eGFP-expressing vectors.

### 3.7. An Ad Prime Followed by Boosting with VSV Led to Massive T Cell Responses to eGFP

**Figure 7:**
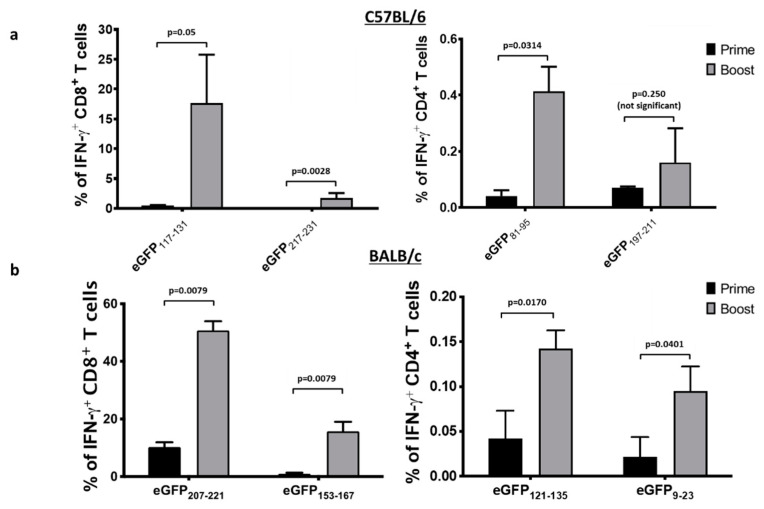
Prime-boosting against eGFP with Ad-eGFP and VSV-eGFP induced CD4^+^ and CD8^+^ T cell responses of high magnitude. (**a**) C57BL/6 and (**b**) BALB/c mice were vaccinated against eGFP by priming intramuscularly with Ad-eGFP at 1 × 10^8^ ifu, and primary responses were assessed 10 days later. Mice were boosted intravenously with VSV-eGFP (1 × 10^9^ pfu) 14 days post-Ad, and the secondary responses were assessed five days post-VSV. Graphs depict CD8^+^ (left) and CD4^+^ (right) T cell responses to the immunodominant and subdominant epitopes of eGFP via intracellular cytokine staining after peptide re-stimulation. Standard errors of the means are shown. Data represent *n* = 3 and *n* = 4 C57BL/6 and BALB/c mice per group, respectively.

With eGFP epitopes completely mapped, we evaluated the magnitude of the primary and secondary immune responses to eGFP using our vaccine vectors. C57BL/6 and BALB/c mice were primed with Ad48-eGFP and boosted with VSV-eGFP. Blood samples were taken at the expected peaks of the primary and secondary T cell responses. Cells were re-stimulated with the immunodominant and subdominant epitopes for each strain of mouse. We were able to achieve a robust mean response of 17.65% and 1.69% of circulating CD8^+^ cells specific for the C57BL/6 dominant (eGFP_117–131_) and subdominant (eGFP_217–231_) eGFP peptides, respectively (Figure 7a). The BALB/c mice had mean secondary CD8^+^ T cell responses of 50.78% and 15.7% to the dominant (eGFP_207–221_) and subdominant (eGFP_9–23_) eGFP epitopes, respectively (Figure 7b). Each strain had prominent CD4^+^ responses as well (Figure 7). Notably, the primary responses to Ad-eGFP alone were insufficient to bring the responses above our detection threshold for the BALB/c CD4^+^ epitopes. Cumulatively, our data suggested that the adenovirus prime and rhabdovirus boost strategy could induce extremely robust responses and unveil novel epitopes, even in the context of a protein of relatively low immunogenicity, such as eGFP. These findings suggest the Ad/VSV prime-boost platform is useful for highly sensitive epitope mapping. They also provide further evidence that the absence of survivin-specific T cell responses using this platform was likely due to extremely robust immunological tolerance to survivin.

## 4. Discussion

We previously described a viral vectored, heterologous rapid prime-boost vaccine platform that induced potent tumor antigen-specific immune responses [8]. This platform was able to generate massive T cell responses by using a replication-deficient adenovirus expressing a transgene under a strong promoter to bias primary T responses towards the transgene rather than the viral backbone proteins. These T cell responses could then be efficiently boosted at very short intervals due to the unique tropism for rhabdoviruses to target privileged antigen presentation sites in splenic B cell follicles, where antigen-presenting cells co-localize with memory T cell cells and are relatively isolated from effector T cells [17]. Our previous DCT-expressing vectors had a narrow indication for treating cancers, as expression of the target antigen was limited to melanomas. As such, we sought out a new tumor antigen with a broader range of expression among cancer types. To this end, we selected survivin based on its high ranking among potentially ideal candidate tumor antigens [2] due to its abundant expression in a wide variety of tumors and relatively low expression in most normal adult tissues [46]. Therefore, a transgene encoding survivin was cloned into our viral vectors. An adenovirus and Maraba virus expressing a tagged, inactivated mutant version of full-length murine survivin were rescued. Accurate engineering of the vectors was confirmed by Western blots that demonstrated expression of survivin in infected cells. Using an overlapping peptide library, we were unable to detect survivin-specific T cell responses after peptide re-stimulation in mice that had been primed and boosted with these vectors. This was a surprising finding, considering two groups were able to demonstrate a survivin-specific T cell response using the mSurvivin_53–67_ peptide [12,13]. Both groups conducted MHC class I binding assays and found this peptide was able to bind, although one group used a smaller peptide within the 15-mer (mSurvivin_57–64_ [CFFCFKEL]). Both groups used peptide-pulsed dendritic cell vaccines, which contrasted with our use of a viral-vectored platform. In our hands, Ad vectors typically induce robust T cell responses against encoded transgenes. Therefore, an inability to do this against survivin led us to conduct experiments to troubleshoot this issue.

An in vivo cytotoxicity assay was conducted (Figure 2), utilizing the mSurvivin_53–67_ peptide to label cells as potential targets for survivin-specific CTLs. However, the results of this assay inferred there were no substantial survivin-specific T cell responses since there was no lysis of the labelled target cells. The assay worked because mice vaccinated in parallel against SIINFEKL had evidence of dramatic killing of SIINFEKL peptide-pulsed target cells. In the past, our heterologous prime-boost platform generated unprecedented T cell responses, even when targeting poorly immunogenic self-derived epitopes. To confirm these historical findings, the Ad-prime/oncolytic virus boost strategy was used to target the relatively poorly immunogenic antigen eGFP. In agreement with historical results, eGFP-specific T cell responses of unprecedented magnitudes could be induced in two strains of mice that are known to be at opposite ends of the T_helper_1 versus T_helper_2 spectrum of immunological bias (Figure 7). Therefore, as an alternate explanation for why survivin-specific responses were not detected, it was hypothesized, based on a previous publication, that survivin-specific T cells were being killed, via a mechanism known as fratricide, due to upregulation of survivin following T cell activation. This fratricidal effect had previously been reported in survivin-specific T cells with transgenic high-avidity TCRs. The authors speculated that such an effect may have consequences for the development of vaccine-induced responses targeting surviving [31]. However, this claim was disputed by one group that did not observe such an effect when using a peptide-pulsed DC-based vaccine [37]. To provide additional commentary to this issue, we co-vaccinated mice with Ad-DCT and Ad-T34A-mSurvivin simultaneously, with controls receiving the Ad-DCT vaccine alone. Compared to single-vaccinated controls, abrogation of DCT-specific T cell responses did not occur. Since the two vaccines were injected in the same bolus, activation of DCT-specific T cells should have occurred in the same lymph nodes as where the survivin-specific T cell response should have developed. In conjunction with the previous publication arguing against fratricide, this result provides strong evidence that this was not the reason for being unable to induce detectable survivin-specific T cell responses.

T cell self-presentation of abundant antigens to high-avidity T cells can induce anergy [47]. We suspected that this might be a mechanism by which survivin-specific T cells could be regulated. CTLA4 plays a pivotal role in the induction of anergy [48], so we reasoned that blocking CTLA4 prior to and concurrent with vaccination could overcome mechanisms of peripheral tolerance. However, this was not able to rescue the lack of a vaccine-induced response, as shown in Figure 5. Several groups have begun human clinical trials targeting human survivin using peptide mimics, which substitute amino acids to enhance binding to MHC molecules in various HLA haplotypes, suggesting naturally derived peptides lacked immunogenicity due to an inability to be presented to T cells [10,38]. However, another group found several naturally occurring survivin epitopes in humanized mice without the need for peptide mimics, calling into question the necessity of mimics [49]. Although our vaccines targeted murine survivin, it is doubtful that poor binding to MHC fully explains the lack of induction of a T cell response. This is because two independent groups elicited a response using the mSurvivin_53–67_ peptide in C57BL/6 mice. It is possible that during the induction of a survivin-specific T cell response, these cells underwent apoptosis through mechanisms such as Fas ligation or TNF signaling [50]. Given that other groups successfully developed peptide-pulsed vaccines using dendritic cells, perhaps there is unique biology to the use of a viral vector versus autologous cells that warrants further exploration. A viral construct could conceivably create a “suicidal” milieu in a vaccine-draining lymph node for autoreactive T cells by inducing substantial quantities of TNF or Fas ligand. An alternative explanation for the difference in the two vaccine platforms could be related to antigen processing. Using a viral vector to get survivin-derived peptides expressed by MHC requires processing of the protein. In contrast, DC-based vaccines bypass this requirement. Finally, it is theoretically possible that full-length survivin contains tolerogenic or immunosuppressive epitopes that are absent when only targeting the mSurvivin_53–67_ peptide. These ideas would be worth exploring further to better understand how to engineer an ideal vaccine against survivin. Regardless, our data suggest that breaking immunological tolerance against full-length survivin may not be feasible with virus-vectored vaccines. A caveat of this study is that only female mice were assessed. Although it would be highly unusual for there to be a sex-specific limitation of the immunogenicity of survivin, this cannot be definitively ruled out. This may warrant repeating the research with males in the future.

The discovery of eGFP has been an incredibly valuable tool for researchers in various fields of study. Indeed, there are a plethora of cancer models that use eGFP-expressing cell lines [51]. The evaluation of eGFP-specific T cell responses is a helpful proof-of-concept tool. Here we expanded on the utility of GFP in immunotherapy by providing researchers with a complete enhanced epitope map of eGFP, which included the discovery of two and three novel CD8^+^ and CD4^+^ T cell epitopes, respectively, across two commonly used strains of mice. Further, this epitope mapping provided confirmation of previously reported immunodominant CD8^+^ T cell epitopes. We also demonstrated that an adenovirus prime followed by a rhabdovirus boost could induce massive eGFP-specific T cell responses in under 20 days. The T cell responses shown in Figure 7 demonstrated that the Ad-prime/rhabdovirus-boost strategy could overcome the relatively poor immunogenicity of eGFP, as had been reported previously [40]. The previous publication described the immunodominant cytotoxic T lymphocyte epitope in C57BL/6 mice, primed with GFP peptides with incomplete Freund’s adjuvant intravenously, and boosted with peptide-pulsed DCs. This yielded a maximum response of ~0.2% responding CD8^+^ T cells from the spleen [43]. In comparison, our prime-boost strategy induced responses of ~17% in the same strain. Our results not only agree with the reported peptides being immunodominant, but they also support the concept that the magnitude of the cytotoxic T lymphocyte response in C57BL/6 mice was less than that of the BALB/c strain. Most importantly, the prime-boost strategy unveiled five novel epitopes in C57BL/6 and BALB/c mice. Subdominant epitopes have been implicated to have important roles in the outcome of anti-tumor immunity. Notably, it has been documented that CD8^+^ T cell responses to dominant epitopes can abrogate the immunogenicity of a tumor if the dominance targets an irrelevant antigen [52]. Moreover, a recent study showed that CD8^+^ T cell responses to dominant epitopes are weakened as a consequence of aging [53]. Such findings highlight the potential utility of effective strategies that can uncover novel sub-dominant epitopes. Therefore, although the vaccine strategy proved unsuccessful in the context of targeting survivin for the treatment of cancers, the studies reported here revealed its novel potential for high resolution epitope mapping.

The robust performance of our vaccine platform when targeting eGFP makes us confident in our vaccine technology, but, in light of our survivin findings, leaves us with a cautionary tale in terms of tumor-antigen selection. No substantial strain-related differences were noted in the T cell responses. Survivin-specific T cell responses could not be induced in either strain of mouse. In contrast, eGFP-specific T cells could be induced in both strains. This is notable because C57BL/6 and BALB/c mice represent opposite ends of the immunological bias spectrum, i.e., a bias towards Th1- and Th2-associated immune responses, respectively.

## 5. Conclusions

We utilized a unique heterologous prime-boost vaccination strategy that enables rapid, high-resolution, complete epitope mapping within ~20 days from the first injection. Using this strategy, several novel epitopes were mapped for eGFP in the most commonly used mouse strains, C57BL/6 and BALB/c. Although the immunogenicity of eGFP has been debated, we were able to achieve unprecedented eGFP-specific T cell responses, highlighting the robustness of the vaccination platform. We hoped to apply these findings in a therapeutic cancer context, targeting the high priority tumor antigen target survivin. After extensive characterization, we were unable to elicit detectable survivin-specific T cells. When comparing our findings with the approaches used by other labs targeting survivin in humans, we conclude that there may be natural hindrances in the antigen processing and presentation aspects of survivin-targeted immunotherapy in mice.

## Data Availability

Data are available from the corresponding author following a reasonable request.

## Supplementary Materials

The following are available online at https://www.mdpi.com/article/10.3390/cancers13246270/s1, Figure S1: Additional confirmatory Western blots of murine survivin expression post-infection with recombinant adenovirus and Maraba virus, Figure S2: Intracellular cytokine staining after re-stimulation of CD4^+^ and CD8^+^ T cells with the complete overlapping survivin peptide library, Figure S3: Dot plots of individual BALB/c mice CD4^+^ responses to eGFP_9–23_ peptide following re-stimulation, Figure S4: Heavy and light chain fragments of UC10-4F10-11 hybridoma-derived CTLA4-specific antibody, Table S1: Primers used to clone, mutate, and sequence a full-length murine survivin insert in adenoviral and Maraba virus plasmid vectors, Table S2: Full-length overlapping eGFP peptide library, Table S3: Full-length overlapping murine survivin peptide library.
