# Supplementary material for: Using a Prime-Boost Vaccination Strategy That Proved Effective for High Resolution Epitope Mapping to Characterize the Elusive Immunogenicity of Survivin
Source: Cancers (Basel). 2021 Dec 14;13(24):6270. doi: 10.3390/cancers13246270 (PMC8699342; doi:10.3390/cancers13246270)
Supplement: Supplementary file 1 [file cancers-13-06270-s001.zip › cancers-1465405-supplementary.pdf]

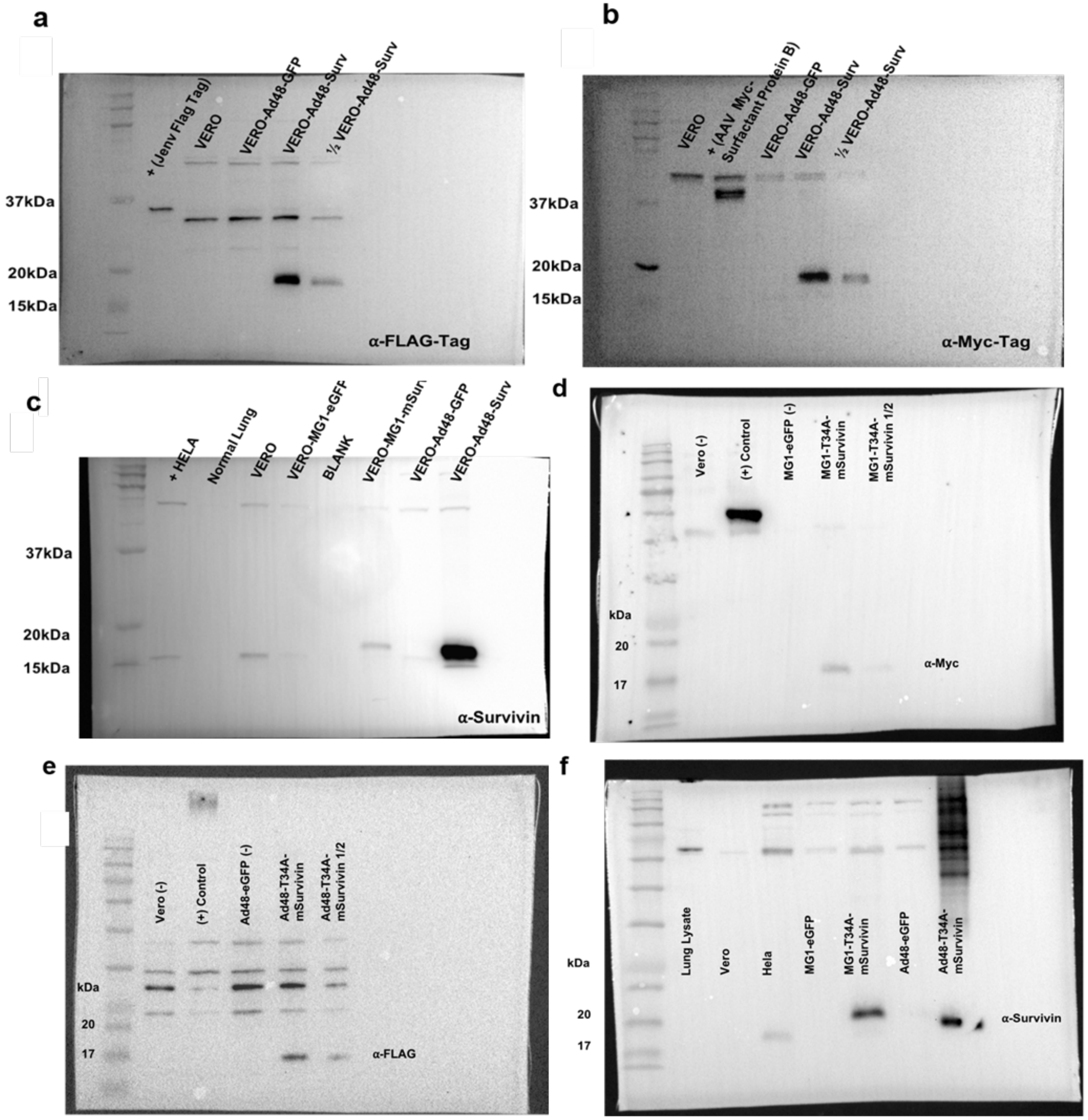
a
+ (Jenv Flag Tag)
VERO
VERO-Ad48-GFP
VERO-Ad48-Surv
½ VERO-Ad48-Surv
37kDa
20kDa
15kDa
α-FLAG-Tag
b
VERO
+ (AAV Myc-Surfactant Protein B)
VERO-Ad48-GFP
VERO-Ad48-Surv
½ VERO-Ad48-Surv
37kDa
20kDa
15kDa
α-Myc-Tag
c
+ HELA
Normal Lung
VERO
VERO-MG1-eGFP
BLANK
VERO-MG1-mSurv
VERO-Ad48-GFP
VERO-Ad48-Surv
37kDa
20kDa
15kDa
α-Survivin
d
Vero (-)
(+) Control
MG1-eGFP (-)
MG1-T34A-mSurvivin
MG1-T34A-mSurvivin 1/2
kDa
20
17
α-Myc
e
Vero (-)
(+) Control
Ad48-eGFP (-)
Ad48-T34A-mSurvivin
Ad48-T34A-mSurvivin 1/2
kDa
20
17
α-FLAG
f
Lung Lysate
Vero
Hela
MG1-eGFP
MG1-T34A-mSurvivin
Ad48-eGFP
Ad48-T34A-mSurvivin
kDa
20
17
α-Survivin

Supplementary Figure S1: Confirmatory western blots of murine survivin expression post-infection with recombinant viruses. Lysates from Vero cells infected with recombinant replication-deficient human adenovirus serotype 48 (Ad48) or replication-competent Maraba virus (MG1) vectors were collected 24 hours later and used to detect the expression of transgene-derived survivin. All lanes were loaded with 15 µg of lysate and probed with a FLAG tag-specific (a,e), Myc-tag-specific (b,d) or survivin-specific antibody (c,f). Lysates from uninfected Vero cells or Vero cells infected with Ad48 or MG1 carrying transgenes encoding for enhanced green fluorescent protein were used as negative controls lacking the expression of tagged-survivin. Normal lung-derived lysate was used as a negative control that did not express substantial amounts of endogenous survivin. FLAG-tagged Jaagsiekte sheep retrovirus enveloped protein (Jenv) was used as a FLAG positive control (a). Myc-tagged surfactant protein B expressed from an adeno-associated virus (AAV) was used as a positive control for Myc (b). Lysate from HeLa cells was used as a positive control for expression of endogenous survivin (c). The tagged murine survivin was expected to be ~5.6% larger than the endogenous survivin protein.

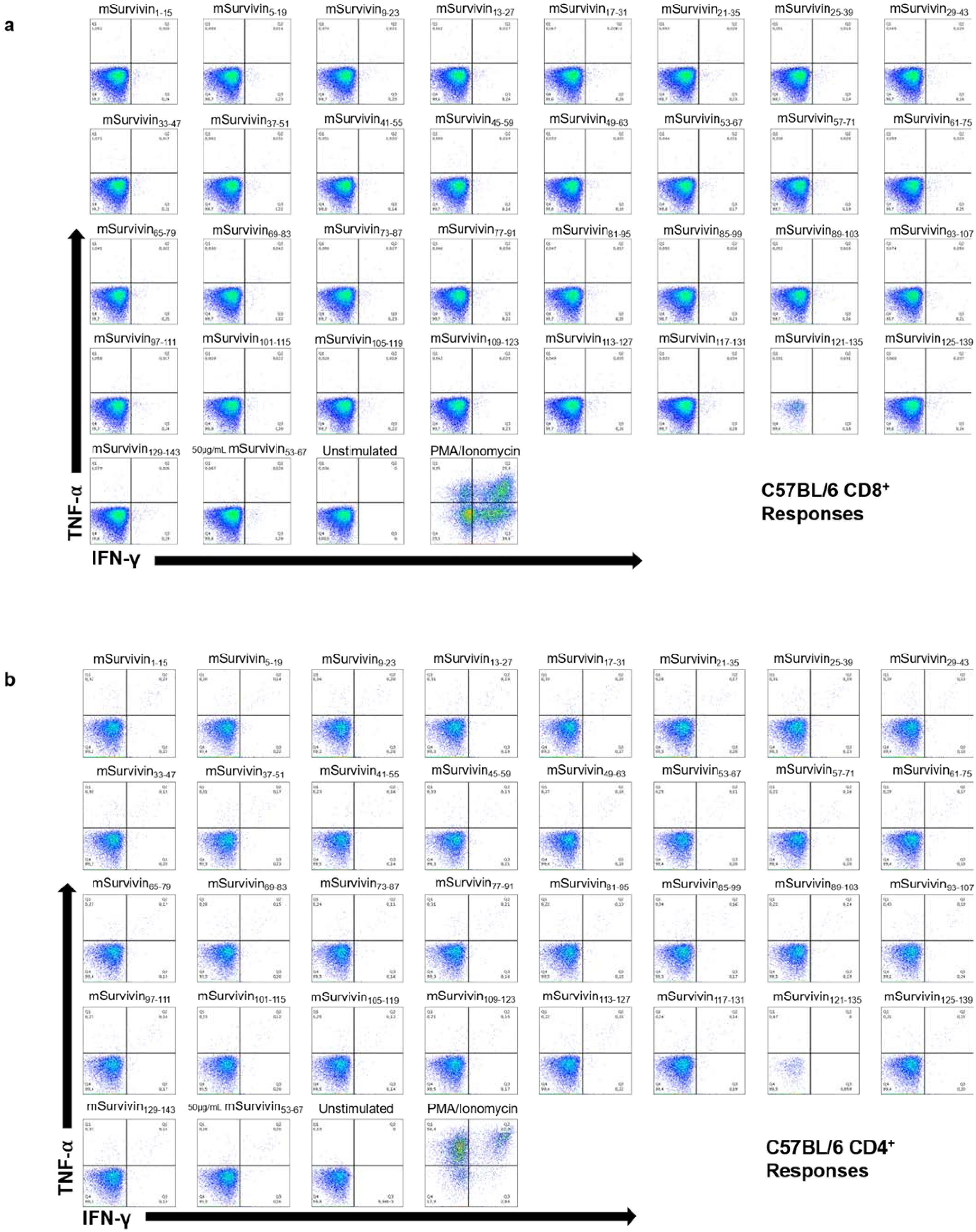
a
mSurvivin1-15
mSurvivin5-19
mSurvivin9-23
mSurvivin13-27
mSurvivin17-31
mSurvivin21-35
mSurvivin25-39
mSurvivin29-43
mSurvivin33-47
mSurvivin37-51
mSurvivin41-55
mSurvivin45-59
mSurvivin49-63
mSurvivin53-67
mSurvivin57-71
mSurvivin61-75
mSurvivin65-79
mSurvivin69-83
mSurvivin73-87
mSurvivin77-91
mSurvivin81-95
mSurvivin85-99
mSurvivin89-103
mSurvivin93-107
mSurvivin97-111
mSurvivin101-115
mSurvivin105-119
mSurvivin109-123
mSurvivin113-127
mSurvivin117-131
mSurvivin121-135
mSurvivin125-139
mSurvivin129-143
50µg/mL mSurvivin53-67
Unstimulated
PMA/Ionomycin
TNF-α
IFN-γ
C57BL/6 CD8+ Responses
b
mSurvivin1-15
mSurvivin5-19
mSurvivin9-23
mSurvivin13-27
mSurvivin17-31
mSurvivin21-35
mSurvivin25-39
mSurvivin29-43
mSurvivin33-47
mSurvivin37-51
mSurvivin41-55
mSurvivin45-59
mSurvivin49-63
mSurvivin53-67
mSurvivin57-71
mSurvivin61-75
mSurvivin65-79
mSurvivin69-83
mSurvivin73-87
mSurvivin77-91
mSurvivin81-95
mSurvivin85-99
mSurvivin89-103
mSurvivin93-107
mSurvivin97-111
mSurvivin101-115
mSurvivin105-119
mSurvivin109-123
mSurvivin113-127
mSurvivin117-131
mSurvivin121-135
mSurvivin125-139
mSurvivin129-143
50µg/mL mSurvivin53-67
Unstimulated
PMA/Ionomycin
TNF-α
IFN-γ
C57BL/6 CD4+ Responses

c

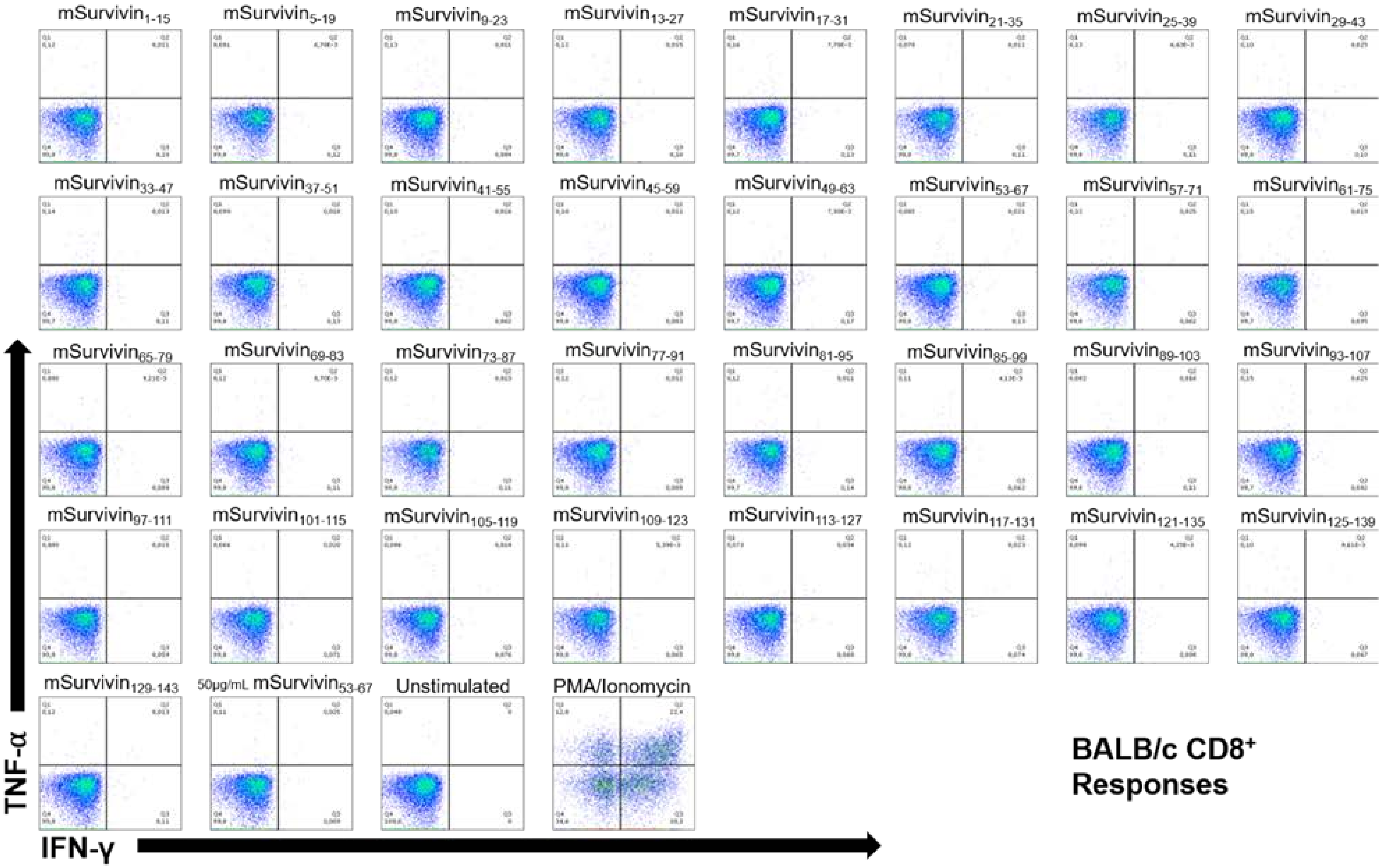

mSurvivin$_{1-15}$
mSurvivin$_{5-19}$
mSurvivin$_{9-23}$
mSurvivin$_{13-27}$
mSurvivin$_{17-31}$
mSurvivin$_{21-35}$
mSurvivin$_{25-39}$
mSurvivin$_{29-43}$
mSurvivin$_{33-47}$
mSurvivin$_{37-51}$
mSurvivin$_{41-55}$
mSurvivin$_{45-59}$
mSurvivin$_{49-63}$
mSurvivin$_{53-67}$
mSurvivin$_{57-71}$
mSurvivin$_{61-75}$
mSurvivin$_{65-79}$
mSurvivin$_{69-83}$
mSurvivin$_{73-87}$
mSurvivin$_{77-91}$
mSurvivin$_{81-95}$
mSurvivin$_{85-99}$
mSurvivin$_{89-103}$
mSurvivin$_{93-107}$
mSurvivin$_{97-111}$
mSurvivin$_{101-115}$
mSurvivin$_{105-119}$
mSurvivin$_{109-123}$
mSurvivin$_{113-127}$
mSurvivin$_{117-131}$
mSurvivin$_{121-135}$
mSurvivin$_{125-139}$
mSurvivin$_{129-143}$
50μg/mL mSurvivin$_{53-67}$
Unstimulated
PMA/Ionomycin
TNF-α
IFN-γ
BALB/c CD8^{+} Responses

d

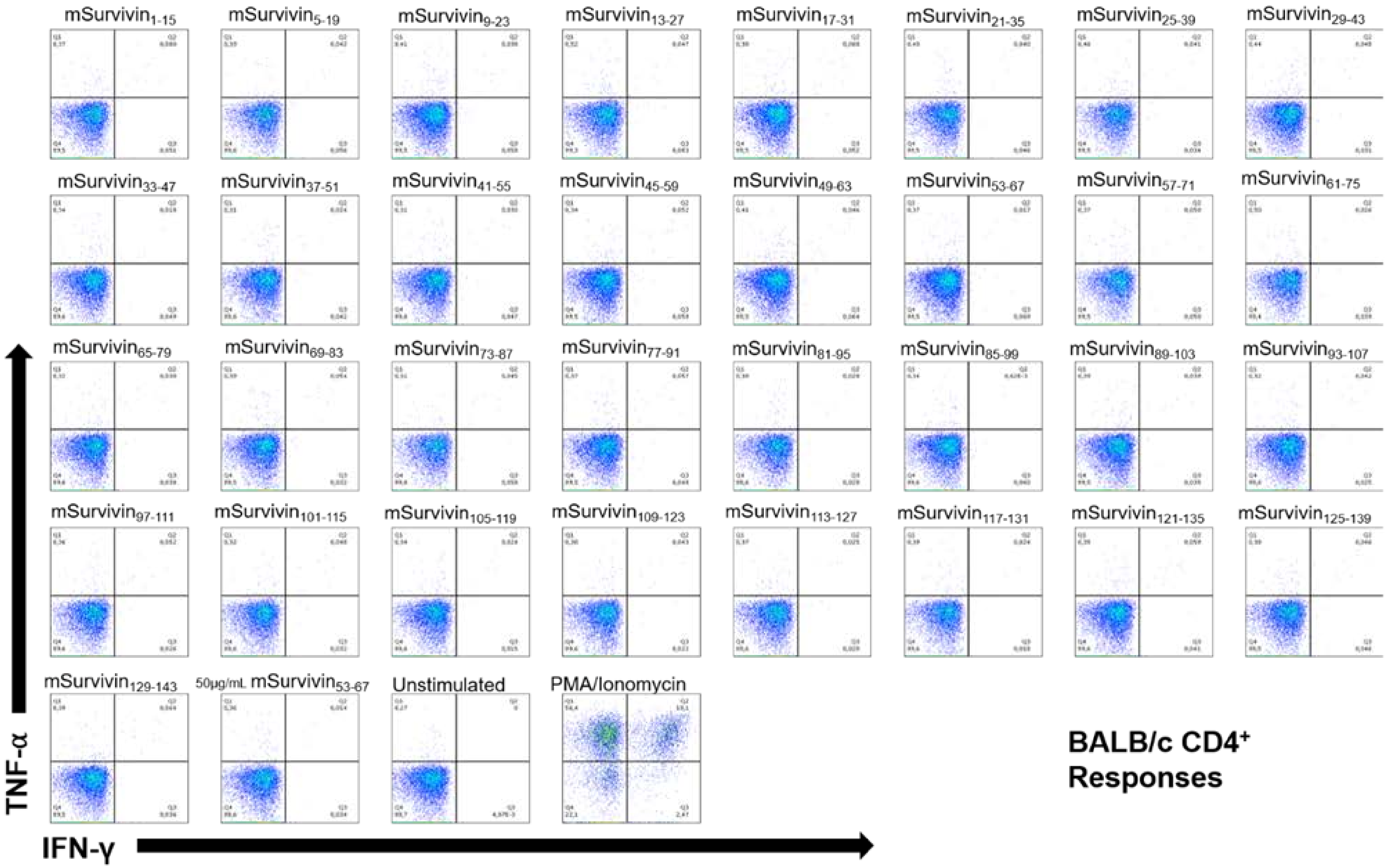

mSurvivin$_{1-15}$
mSurvivin$_{5-19}$
mSurvivin$_{9-23}$
mSurvivin$_{13-27}$
mSurvivin$_{17-31}$
mSurvivin$_{21-35}$
mSurvivin$_{25-39}$
mSurvivin$_{29-43}$
mSurvivin$_{33-47}$
mSurvivin$_{37-51}$
mSurvivin$_{41-55}$
mSurvivin$_{45-59}$
mSurvivin$_{49-63}$
mSurvivin$_{53-67}$
mSurvivin$_{57-71}$
mSurvivin$_{61-75}$
mSurvivin$_{65-79}$
mSurvivin$_{69-83}$
mSurvivin$_{73-87}$
mSurvivin$_{77-91}$
mSurvivin$_{81-95}$
mSurvivin$_{85-99}$
mSurvivin$_{89-103}$
mSurvivin$_{93-107}$
mSurvivin$_{97-111}$
mSurvivin$_{101-115}$
mSurvivin$_{105-119}$
mSurvivin$_{109-123}$
mSurvivin$_{113-127}$
mSurvivin$_{117-131}$
mSurvivin$_{121-135}$
mSurvivin$_{125-139}$
mSurvivin$_{129-143}$
50μg/mL mSurvivin$_{53-67}$
Unstimulated
PMA/Ionomycin
TNF-α
IFN-γ
BALB/c CD4^{+} Responses

Supplementary Figure S2: Intracellular cytokine staining after re-stimulation of $CD4^+$ and $CD8^+$ T cells with the complete overlapping survivin peptide library.

C57BL/6 and BALB/c mice were vaccinated with $1x10^8$ infectious units of Ad-T34A-mSurvivin, then boosted 14 days later with $1x10^9$ plaque-forming units of MG1-T34A-mSurvivin. Spleen samples were taken from vaccinated mice at the peak of the secondary response (day 21), processed and re-stimulated with the peptide indicated above each dot plot for five hours. Panels (a) and (b) indicate $CD8^+$ and $CD4^+$ T cell responses respectively, demonstrated by staining for intracellular interferon gamma (IFN-y) and tumor necrosis factor alpha (TNF-α) in C57BL/6 mice using flow cytometry. Similarly, $CD8^+$ and $CD4^+$ T cell responses are respectively shown in panels (c) and (d) in BALB/c mice. Data shown are representative dot plots from one of three mice of each strain. Non-specific stimulation with phorbol 12-myristate 13-acetate (PMA) and ionomycin was used as a positive control to demonstrate that the assay worked.

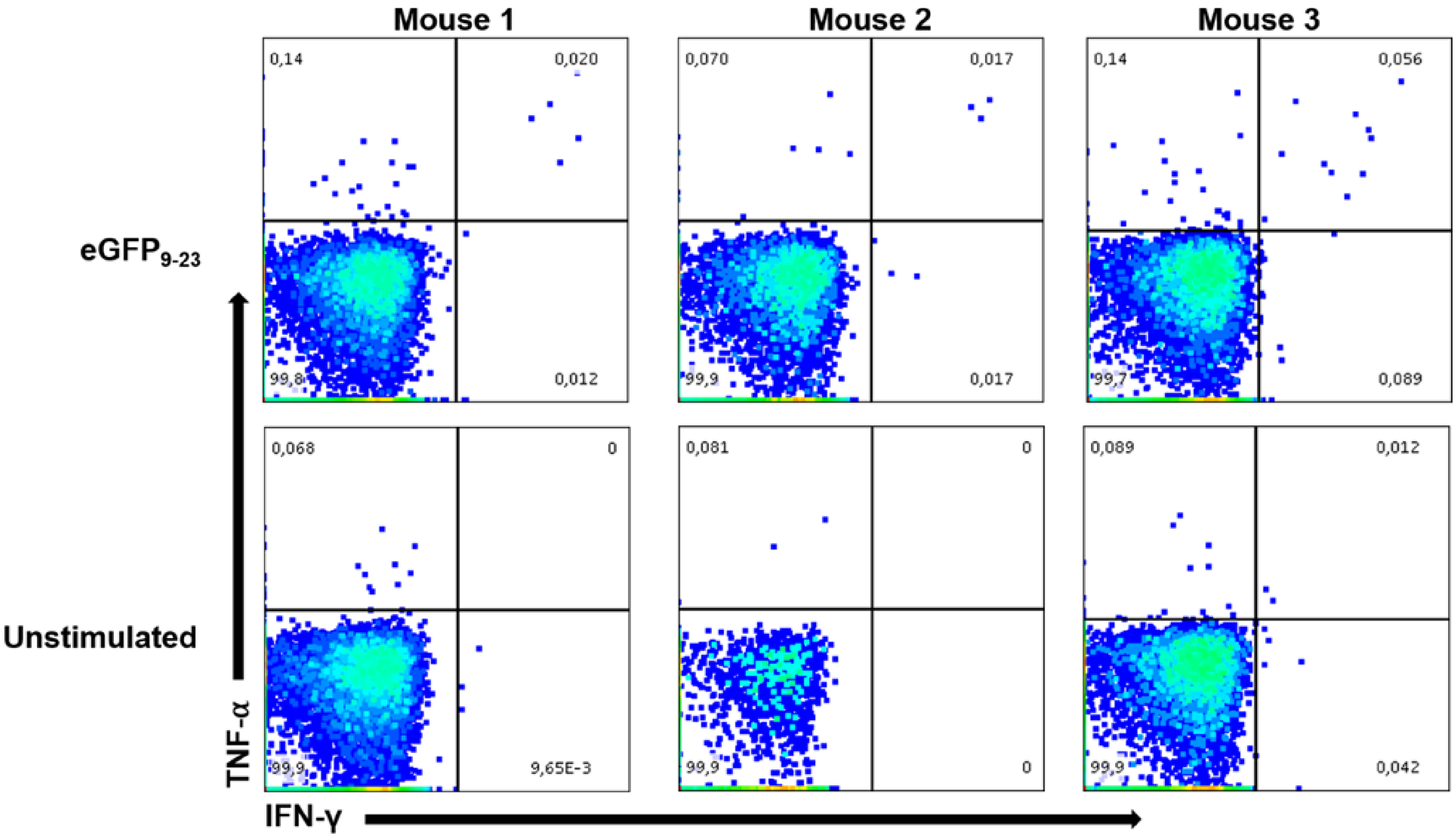

Supplementary Figure S3: Flow cytometry-derived dot plots from three BALB/c mice demonstrate $CD4^+$ responses to the enhanced green fluorescent protein $(eGFP)_{9\text{-}23}$ peptide.

Mice were vaccinated intramuscularly with $1x10^8$ infectious units of a recombinant replication-deficient human adenovirus serotype 48 carrying a transgene encoding enhanced green fluorescent protein. Fourteen days later the mice were boosted with a recombinant replication-competent Maraba virus carrying a transgene encoding enhanced green fluorescent protein. Five days post-boost splenocytes were intracellularly stained for interferon gamma (IFN-γ) and tumor necrosis factor alpha (TNF-α) following re-stimulation with the peptide and assessed by flow cytometry. The appearance of events in the upper right quadrant, which shows cells expressing both IFN-γ and TNF-α, in the stimulated samples is suggestive of a real, albeit low-magnitude response.

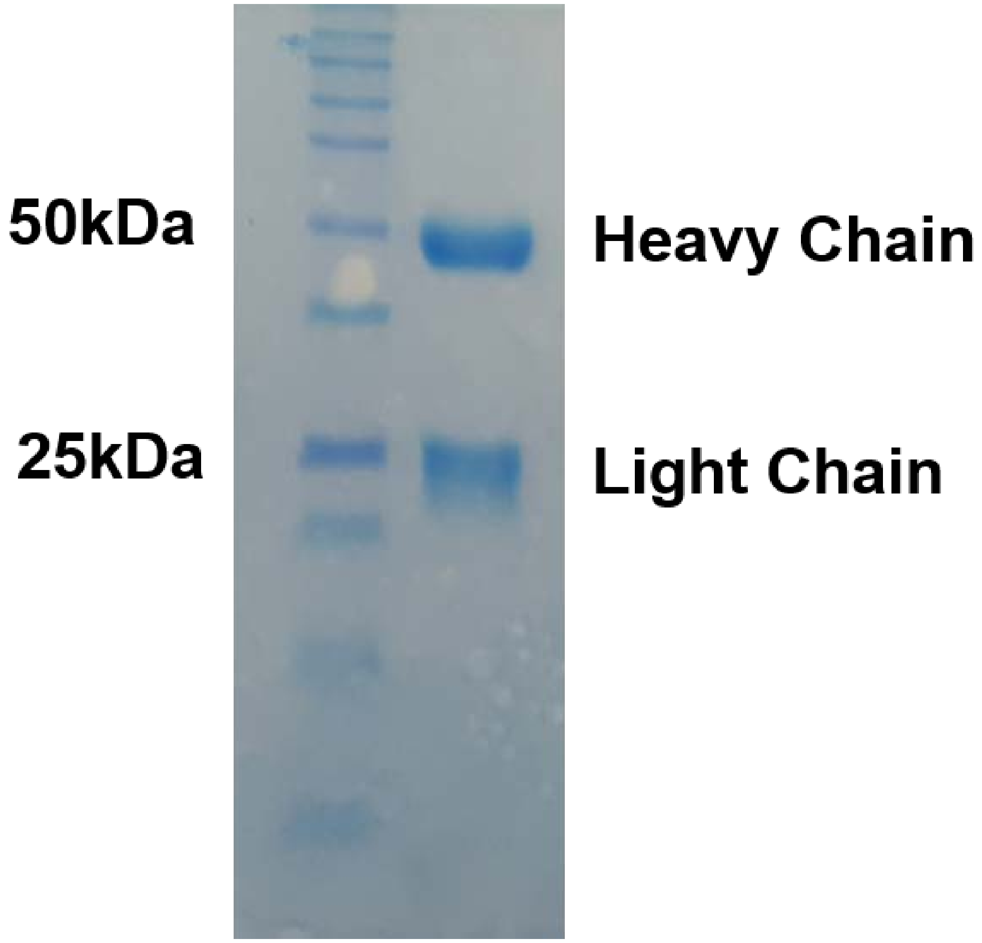

Supplementary Figure S4: Heavy and light chain fragments of UC10-4F10-11 hybridoma-derived anti-cytotoxic T lymphocyte antigen-4 (CTLA4). Four µg of purified CTLA4-specific antibody was denatured, resolved by 12% sodium dodecyl sulphate–polyacrylamide gel electrophoresis, and stained with Coomassie Blue.

Name:	Sequence 5' to 3':
**(Ad) FWD KpnI FLAG-Tag mSurvivin:**	*TATA* GGTACC ACC ATG **GACTACAAGGACGACGACGACAAG** GGAGCTCCGGCGCTGCC
**(Ad) RVS BamHI mSurvivin:**	*TATA* GGATCC TTAGGCAGCCAGCTGCTCAATTGACTG
**(MG1) FWD SalI Myc-Tag mSurvivin:**	*TATA* GTCGAC ACC ATG **GAGCAGAAGCTTATCTCCGAGGAGGACCTT** GGAGCTCCGGCGCTGCC
**(MG1) RVS SpeI mSurvivin:**	*TATA* ACTAGT TTAGGCAGCCAGCTGCTCAATTGACTG
**AdApt48CAGseq FWD:**	CGGCTCTAGAGCCTCTGCTAACCATG
**AdApt48CAGseq RVS:**	GGACAAACCACAACTAGAATGCAGTG
**MG1insrtSeq FWD:**	CGATTGGGAAATAAATAACAGATGACGCATG
**MG1insrtSeq RVS:**	CATTTGGTCTTCCGGATTGAGAAATTCTG
**mSurv-T34A-SDM FWD:**	GGAGGACTGCGCCTGCGCCCCAGAGCGAATGGCGG
**mSurv-T34A-SDM RVS:**	CCGCCATTCGCTCTGGGGCGCAGGCGCAGTCCTCC

Supplementary Table S1: Primers used to clone, mutate, and sequence a full-length murine survivin insert in adenoviral and Maraba virus plasmid vectors. The forward primers for cloning survivin had added restriction enzyme cleavage sites (underlined), followed by a Kozak sequence (double underlined) for improved translation initiation, the ATG start codon followed by a FLAG-tag, or Myc-tag sequence for the adenovirus vector and Maraba vector, respectively (bolded). Finally, the primers had 17 base-pairs of homology to murine survivin. Similarly, the reverse primers had a restriction enzyme cleavage site and 27-base-pairs of homology. The sequencing primers for the adenovirus vector and Maraba vector had homology to the flanking regions of the insert portion of the pAdApt and pMG1-Genome plasmids, respectively. The site-directed-mutagenesis (SDM) primers were homologous for murine survivin, with the exception of the mutation site (double underlined).

Supplementary Table S2: Full-length overlapping (by 12 amino acids) enhanced green fluorescent protein 15-mer peptide library.

Peptide	Peptide Sequence
eGFP$_{1-15}$	MVSKGEELFTGVVPI
eGFP$_{5-19}$	GEELFTGVVPILVEL
eGFP$_{9-23}$	FTGVVPILVELDGDV
eGFP$_{13-27}$	VPILVELDGDVNGHK
eGFP$_{17-31}$	VELDGDVNGHKFSVS
eGFP$_{21-35}$	GDVNGHKFSVSGEGE
eGFP$_{25-39}$	GHKFSVSGEGEGDAT
eGFP$_{29-43}$	SVSGEGEGDATYGKL
eGFP$_{33-47}$	EGEGDATYGKLTLKF
eGFP$_{37-51}$	DATYGKLTLKFICTT
eGFP$_{41-55}$	GKLTLKFICTTGKLP
eGFP$_{45-59}$	LKFICTTGKLPVPWP
eGFP$_{49-63}$	CTTGKLPVPWPTLVT
eGFP$_{53-67}$	KLPVPWPTLVTTLTY
eGFP$_{57-71}$	PWPTLVTTLTYGVQC
eGFP$_{61-75}$	LVTTLTYGVQCFSRY
eGFP$_{65-79}$	LTYGVQCFSRYPDHM
eGFP$_{69-83}$	VQCFSRYPDHMKQHD
eGFP$_{73-87}$	SRYPDHMKQHDFFKS
eGFP$_{77-91}$	DHMKQHDFFKSAMPE
eGFP$_{81-95}$	QHDFFKSAMPEGYVQ
eGFP$_{85-99}$	FKSAMPEGYVQERTI
eGFP$_{89-103}$	MPEGYVQERTIFFKD
eGFP$_{93-107}$	YVQERTIFFKDDGNY
eGFP$_{97-111}$	RTIFFKDDGNYKTRA
eGFP$_{101-115}$	FKDDGNYKTRAEVKF
eGFP$_{105-119}$	GNYKTRAEVKFEGDT
eGFP$_{109-123}$	TRAEVKFEGDTLVNR
eGFP$_{113-127}$	VKFEGDTLVNRIELK
eGFP$_{117-131}$	GDTLVNRIELKGIDF
eGFP$_{121-135}$	VNRIELKGIDFKEDG
eGFP$_{125-139}$	ELKGIDFKEDGNILG
eGFP$_{129-143}$	IDFKEDGNILGHKLE
eGFP$_{133-147}$	EDGNILGHKLEYNYN
eGFP$_{137-151}$	ILGHKLEYNYNSHNV
eGFP$_{141-155}$	KLEYNYNSHNVYIMA
eGFP$_{145-159}$	NYNSHNVYIMADKQK
eGFP$_{149-163}$	HNVYIMADKQKNGIK
eGFP$_{153-167}$	IMADKQKNGIKVNFK
eGFP$_{157-171}$	KQKNGIKVNFKIRHN
eGFP$_{161-175}$	GIKVNFKIRHNIEDG
eGFP$_{165-179}$	NFKIRHNIEDGSVQL
eGFP$_{169-183}$	RHNIEDGSVQLADHY
eGFP$_{173-187}$	EDGSVQLADHYQQNT
eGFP$_{177-191}$	VQLADHYQQNTPIGD
eGFP$_{181-195}$	DHYQQNTPIGDGPVL
eGFP$_{185-199}$	QNTPIGDGPVLLPDN
eGFP$_{189-203}$	IGDGPVLLPDNHYLS
eGFP$_{193-207}$	PVLLPDNHYLSTQSA
eGFP$_{197-211}$	PDNHYLSTQSALSKD
eGFP$_{201-215}$	YLSTQSALSKDPNEK
eGFP$_{205-219}$	QSALSKDPNEKRDHM
eGFP$_{209-223}$	SKDPNEKRDHMVLLE
eGFP$_{213-227}$	NEKRDHMVLLEFVTA
eGFP$_{217-231}$	DHMVLLEFVTAAGIT
eGFP$_{221-235}$	LLEFVTAAGITLGMD
eGFP$_{225-239}$	VTAAGITLGMDELYK
eGFP$_{229-239}$	GITLGMDELYK

Supplementary Table S3: Full-length overlapping (by 12 amino acids) murine survivin 15-mer peptide library.

Peptide	Peptide Sequence
$mSurvivin_{1-15}$	MGAPALPQIWQLYLK
$mSurvivin_{5-19}$	ALPQIWQLYLKNYRI
$mSurvivin_{9-23}$	IWQLYLKNYRIATFK
$mSurvivin_{13-27}$	YLKNYRIATFKNWPF
$mSurvivin_{17-31}$	YRIATFKNWPFLEDC
$mSurvivin_{21-35}$	TFKNWPFLEDCACTP
$mSurvivin_{25-39}$	WPFLEDCACTPERMA
$mSurvivin_{29-43}$	EDCACTPERMAEAGF
$mSurvivin_{33-47}$	CTPERMAEAGFIHCP
$mSurvivin_{37-51}$	RMAEAGFIHCPTENE
$mSurvivin_{41-55}$	AGFIHCPTENEPDLA
$mSurvivin_{45-59}$	HCPTENEPDLAQCFF
$mSurvivin_{49-63}$	ENEPDLAQCFFCFKE
$mSurvivin_{53-67}$	DLAQCFFCFKELEGW
$mSurvivin_{57-71}$	CFFCFKELEGWEPDD
$mSurvivin_{61-75}$	FKELEGWEPDDNPIE
$mSurvivin_{65-79}$	EGWEPDDNPIEEHRK
$mSurvivin_{69-83}$	PDDNPIEEHRKHSPG
$mSurvivin_{73-87}$	PIEEHRKHSPGCAFL
$mSurvivin_{77-91}$	HRKHSPGCAFLTVKK
$mSurvivin_{81-95}$	SPGCAFLTVKKQMEE
$mSurvivin_{85-99}$	AFLTVKKQMEELTVS
$mSurvivin_{89-103}$	VKKQMEELTVSEFLK
$mSurvivin_{93-107}$	MEELTVSEFLKLDRQ
$mSurvivin_{97-111}$	TVSEFLKLDRQRAKN
$mSurvivin_{101-115}$	FLKLDRQRAKNKIAK
$mSurvivin_{105-119}$	DRQRAKNKIAKETNN
$mSurvivin_{109-123}$	AKNKIAKETNNKQKE
$mSurvivin_{113-127}$	IAKETNNKQKEFEET
$mSurvivin_{117-131}$	TNNKQKEFEETAKTT
$mSurvivin_{121-135}$	QKEFEETAKTTRQSI
$mSurvivin_{125-139}$	EETAKTTRQSIEQLA
$mSurvivin_{129-143}$	ETAKTTRQSIEQLAA
